# Effectiveness and Safety of Pretreatment Analgesics and Anti‐Inflammatory Drugs in Endodontic Pain Management: Overview of Systematic Reviews

**DOI:** 10.1155/ijod/7258438

**Published:** 2026-08-09

**Authors:** Cristiane Midori Takahashi, Régis Penha Pimenta, Cristiane de Cássia Bergamaschi, Fabio da Silva Moraes, Silvio Barberato-Filho

**Affiliations:** ^1^ Graduate Program in Pharmaceutical Sciences, University of Sorocaba, Sorocaba, São Paulo, Brazil, uniso.br; ^2^ FIT Institute of Technology, Sorocaba, São Paulo, Brazil

**Keywords:** analgesic, anti-inflammatory, effectiveness, endodontics, pain management, safety

## Abstract

**Background:**

Postoperative pain in endodontic treatment remains a significant challenge and concern for specialists. Despite advancements in treatment techniques, reports of postoperative pain due to trauma to the periapical tissues persist. The pretreatment anti‐inflammatory drugs (nonsteroidal anti‐inflammatory drugs [NSAIDs] and corticosteroids) and analgesic drugs (opioids and nonopioids) are a pharmacotherapeutic strategy employed for pain relief and inflammation control in endodontics.

**Objective:**

This overview of systematic reviews of randomized controlled trials (RCTs) synthesized the available evidence on the effectiveness and safety of pretreatment anti‐inflammatory and analgesic drugs in pain management for patients undergoing endodontic treatment.

**Methods:**

The databases searched included the Cochrane Library, MEDLINE, EMBASE, Epistemonikos, Scopus, Web of Science, and Virtual Health Library, up to March 2023. Pairs of reviewers independently selected the studies, extracted the data, and assessed the methodological quality using the AMSTAR‐2 (A MeaSurement Tool to Assess systematic Reviews 2) tool.

**Results:**

Fourteen systematic reviews that met the eligibility criteria were included, published between 2016 and 2022, involving between 3 and 27 primary studies that addressed pretreatment use of drugs. Six systematic reviews conducted meta‐analyses and found that the pretreatment oral or parenteral corticosteroids reduced postoperative pain, compared with placebo, for 6, 12, and 24 h after nonsurgical endodontic treatment. NSAID systematic reviews have presented many conflicting results. Most reviews were of critically low quality, and data on adverse effects were poorly reported.

**Conclusion:**

Pretreatment corticosteroids appeared to be associated with more consistent reductions in postoperative pain among patients undergoing endodontic treatment. However, confidence in these findings is limited by substantial heterogeneity, the critically low methodological quality of most reviews, and poor reporting of adverse effects. Further high‐quality RCTs are needed.

## 1. Background

Postoperative pain in endodontics is a significant concern for specialists as it can impact the patient’s quality of life and treatment expectations [1, 2]. Research shows 16%–24% of patients experience moderate to severe postoperative pain [3, 4]. Despite advancements in treatment techniques and the use of intracanal medications, postoperative pain still occurs. This can be attributed to trauma inflicted on periapical tissues during treatment or the extrusion of microorganisms and dentinal debris, leading to an acute inflammatory reaction [5].

Studies have shown that the presence of postoperative pain is mainly associated with cases in which there is preoperative pain. A systematic review identified preoperative pain as one of the main predictors of pain after endodontic treatment [6].

A resource used for pain relief and inflammation control in endodontics is the use of systemic medication, which is the subject of investigation through randomized controlled trials (RCTs) to minimize postoperative pain [7, 8]. Among the drugs studied, non‐steroidal anti‐inflammatory drugs (NSAIDs) [9, 10], corticosteroids [11, 12], and opioid analgesics [13] stand out.

Ibuprofen has been the most widely used NSAID due to its low cost, effectiveness, and safety. Some studies cite that a dose of ibuprofen before the procedure is effective in reducing postoperative pain [14, 15]. However, another study reports no significant reduction in pain after endodontic treatment compared to placebo [13, 16].

In patients who report gastric sensitivity, the use of NSAIDs should be avoided as they also act on cyclooxygenase‐1 (COX‐1), inhibiting the synthesis of prostaglandins responsible for gastric protection. Therefore, it is better to prescribe steroid anti‐inflammatories or opioid analgesics. It should be mentioned that this last class of drugs has restricted use due to adverse effects such as dizziness, nausea, respiratory depression, and chemical dependance [17].

A steroid anti‐inflammatory widely used for pretreatment analgesia is dexamethasone as it has a good response in controlling pain, edema, and trismus after dental surgeries [18].

Another important aspect to consider is the number of doses administered. Pretreatment medication, when proven to be effective and safe, holds a significant advantage over the administration of multiple postoperative doses. This approach helps minimize the adverse effects associated with the medications [19, 20].

In endodontics, the distinction between preemptive and preventive analgesia should be interpreted within the specific context of treatment‐related nociceptive and inflammatory events. Preemptive analgesia refers to the administration of an analgesic or anti‐inflammatory intervention before the onset of endodontic procedures, particularly prior to access opening, canal instrumentation, and irrigation, with the aim of attenuating the initial nociceptive input associated with tissue manipulation. Preventive analgesia, in contrast, represents a broader perioperative concept encompassing strategies designed not only to initiate analgesic coverage before treatment but also to maintain modulation of inflammatory and nociceptive pathways during the postoperative period of greatest risk for pain development [21–23].

In this overview, pretreatment refers to the administration of an analgesic or anti‐inflammatory medication before the initiation of the endodontic procedure. This terminology was adopted because, in endodontic practice, many patients present with pre‐existing pain and active inflammation before treatment begins, particularly in cases of symptomatic irreversible pulpitis, in which peripheral nociceptive sensitization may already be established at the time of clinical intervention [22]. Under these conditions, the term preemptive analgesia, classically defined as an intervention administered before tissue injury with the aim of preventing central sensitization and reducing subsequent postoperative pain, may be conceptually less precise when applied to endodontic pain models [16]. Preventive analgesia was not considered in this study because it represents a broader perioperative concept encompassing strategies designed not only to initiate analgesic coverage before treatment but also to maintain modulation of inflammatory and nociceptive pathways during the postoperative period of greatest risk for pain development [21].

Despite the increasing number of randomized clinical trials evaluating pretreatment in endodontics, the available evidence demonstrates substantial methodological heterogeneity. Differences among studies include the pharmacological agents evaluated, dosages, routes of administration, pulpal and periapical conditions, instrumentation protocols, irrigation regimens, and methods used for postoperative pain assessment [6, 21, 23].

Therefore, an overview of systematic reviews is warranted to critically synthesize the current evidence regarding pretreatment in endodontics and assess the methodological consistency of the available evidence on the effectiveness and safety of the pretreatment anti‐inflammatory and analgesic drugs in pain management for patients undergoing nonsurgical endodontic treatment.

## 2. Methods

### 2.1. Study Design and Protocol Registration

This overview of systematic reviews was conducted following the Preferred Reporting Items for Systematic Reviews and Meta‐Analyses (PRISMA) statement and the recommendations of the Cochrane Handbook for Systematic Reviews of Interventions [24]. The protocol was registered in the International Prospective Register of Systematic Reviews (PROSPERO) under protocol CRD42022355785. This overview is part of a broader study on the use of analgesics and anti‐inflammatory agents in dentistry. The search, eligibility determination, and data extraction steps were carried out concurrently with the study by Pimenta et al. [25].

### 2.2. Eligibility Criteria

Eligibility criteria were described using the Population, Intervention, Comparison, Outcome, and Type of Study (PICOT) framework.

#### 2.2.1. Inclusion Criteria

• Population: Patients undergoing root canal treatment in permanent teeth with complete root formation, regardless of pulpal or periapical diagnosis.
• Intervention: Pretreatment with anti‐inflammatory drugs (NSAIDs and corticosteroids) and analgesic drugs (opioids and nonopioids) at any dose.
• Comparator: Placebo or other active control used to manage pain.
• Outcome: Effectiveness (pain relief and inflammation control in endodontics) and safety (adverse drug reactions).
• Type of study: Systematic reviews of RCTs.

#### 2.2.2. Exclusion Criteria

• Intervention: Studies in which the timing of drug administration was uncertain and studies involving the administration of medications after the start of treatment or with intracanal administration of these medicines.
• Type of study: Abstracts presented at scientific events were also excluded.

### 2.3. Evaluated Outcomes

• Primary outcomes: Pain relief and inflammation control in endodontics. The description of symptoms may be by self‐report, validated questionnaires, or clinical diagnosis. Pain may have been assessed using scales such as the visual analogue scale (VAS), Heft‐Parker visual analog scale, numerical rating scale (NRS), or another measure validated by the literature.
• Secondary outcomes: Improved quality of life, analgesic consumption in the postoperative period (rescue medication), and the presence of adverse drug reactions.

### 2.4. Research Sources for Systematic Reviews

#### 2.4.1. Electronic Searches

The databases searched included the Cochrane Library, MEDLINE (via PubMed), EMBASE, Epistemonikos, Scopus, Web of Science, and Virtual Health Library. The search included studies without language restrictions or time limits. We used information sources to locate the studies from the beginning up to March 2023.

#### 2.4.2. Searching for Other Resources

The reference lists of the reviews were checked by the reviewers to identify other possible studies. If necessary, the corresponding authors of the studies were contacted for additional information.

### 2.5. Search Strategy

The search strategy used for the PubMed database, which combined MeSH terms and cross‐referenced synonyms (Entry Terms) related to endodontics, drugs, preventive, and systematic review, is shown in Supporting Information 1: Table S1. This same strategy was adapted for the other databases that were searched.

### 2.6. Eligibility Determination

Pairs of reviewers (including the RPP, CMT, and FSM) independently assessed potentially relevant titles and abstracts and applied eligibility criteria. The full text of systematic reviews was obtained. These reviewers independently assessed the eligibility of each full text and resolved disagreements by consensus. Calibration exercises were performed for data extraction using a standardized Excel form. A third reviewer (CCB or SB‐F) helped with the final decision when necessary.

### 2.7. Methodological Quality Assessment

The methodological quality of systematic reviews was assessed by the same reviewers, in pairs and independently, using the AMSTAR 2 instrument (A MeaSurement Tool to Assess Systematic Reviews 2). This tool assesses the methodological aspects of systematic reviews through 16 items and classifies the overall confidence in the review results as high (no or one noncritical weakness), moderate (more than one non‐critical weakness), low (one critical flaw with or without non‐critical weaknesses), and critically low (more than one critical flaw with or without non‐critical weaknesses) [26]. Disagreements were resolved by consensus among the evaluators. Any unresolved disagreements were referred to a third reviewer (CCB or SB‐F).

### 2.8. Data Extraction

A data extraction spreadsheet was previously developed to record the information collected. Two reviewers (CMT and RPP), in pairs and independently, extracted the data and recorded the information regarding the patients, methods, interventions, risk of bias, outcomes, and absence of important outcomes. Disagreements were resolved by consensus, and any unresolved issues were referred to a third reviewer (CCB or SB‐F).

This extraction was performed in accordance with the instruction manual prepared and pretested by the main author of this review. In the absence of any information, the systematic review author was contacted. In the case of duplicate publication, the review with the most complete data was used.

### 2.9. Data Synthesis

The results of the systematic reviews were summarized through narrative synthesis and are presented in tables. Analyses were provided for each outcome of interest. The summary measures of the findings were described using the odds ratio (OR), relative risk reduction (RRR), standardized mean difference (SMD), mean difference (MD), and weighted mean difference (WMD), followed by 95% confidence intervals (95% CI).

Primary studies in the systematic reviews were reviewed, and those that evaluated the pretreatment anti‐inflammatory and analgesic drugs in the pain management of patients undergoing nonsurgical endodontic treatment were systematized and complemented the analysis.

The measure of heterogeneity in the systematic reviews was collected and described using the *I*‐square (*I* ^2^) statistic, categorized as 0%–25% (low heterogeneity); 50% (moderate heterogeneity); and 75% (high heterogeneity) [27].

The quality of evidence, when available in studies, was collected for each outcome according to Grading of Recommendations Assessment, Development, and Evaluation (GRADE). This tool grades the evidence as high quality (there is confidence that the true effect lies close to that of the estimate of the effect); moderate quality (there is moderate confidence in the effect estimate: the true effect is likely to be close to the estimate of the effect, but there is a possibility that it is substantially different); low quality (the confidence in the effect estimate is limited: the true effect may be substantially different from the estimate of the effect); and very low quality (very little confidence in the effect estimate: the true effect is likely to be substantially different from the estimate of the effect) [28].

To assess overlap among the primary studies included in the systematic reviews, we calculated the corrected covered area (CCA), a method commonly used to quantify evidence redundancy in overviews [29]. A citation matrix was first developed to map the primary studies included in each systematic review. The CCA was then calculated using the formula CCA = (*N*−*r*)/[(*r* × *c*)−*r*], where N is the total number of primary study citations across reviews, *r* is the number of unique primary studies, and *c* is the number of included systematic reviews. CCA values were interpreted according to previously published thresholds: 0%–5% (slight overlap), 6%–10% (moderate overlap), 11%–15% (high overlap), and >15% (very high overlap).

### 2.10. Ethical Aspects

Ethical approval was not required for this overview of systematic reviews as this type of study does not involve the presentation of individual patient data.

## 3. Results

**Figure 1:**
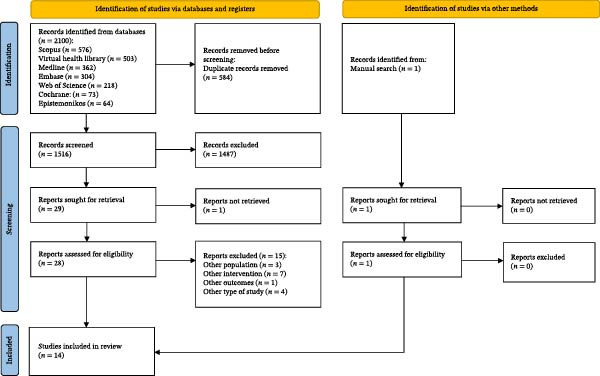
Flowchart of included studies.

A search of the literature initially identified 2100 registers. After deduplication, 1516 records remained. Titles and abstracts were reviewed, and 28 systematic reviews were eligible to be assessed using full‐text retrieval. Additionally, one record was retrieved through a manual search. After this stage, 14 articles were included in this study (Figure 1). The list of excluded studies and reasons for exclusion can be found in Supporting Information 2: Table S2.

### 3.1. Characteristics of Included Studies

The characteristics of the included systematic reviews are presented in Table 1. Six systematic reviews exclusively addressed pretreatment use, while eight reviews addressed both the pretreatment use of drugs and their administration after the beginning of treatment. They were published between 2016 and 2022 and included 3–27 RCTs that addressed pretreatment drugs.

**Table 1:** Characterization of systematic reviews that addressed the pretreatment anti‐inflammatory drugs and analgesics in endodontics (*n* = 14).

Systematic review (search period)	Number of RCT (pretreatment/total)	Age population (years)	Intervention vs comparator (route of administration)	Main objective of review	Conclusions of review based on findings of pretreatment use of drugs
Almuthhin et al. [13] (until Dec/2019)	27/62	14–71 (min–max)	NSAIDs or corticosteroids or COX‐2 inhibitors or opioids vs. placebo (any route)	To summarize current evidence on the efficacy of pre‐ and post‐medication for the treatment of postoperative endodontic pain and rank the available drugs according to their efficacy	Current evidence suggests that pre‐medication can reduce postoperative pain after nonsurgical root canal treatment. However, the authors did not analyze the pretreatment use separately.
Aminoshariae et al. [30] (Jan/1990 to Apr/2016)	9/27	NR	NSAIDs or corticosteroids vs. placebo or other control groups (oral route)	To report the best available scientific evidence regarding the efficacy of analgesics and corticosteroids to decrease endodontic preoperative and postoperative pain	The preoperative administration of corticosteroids was effective in reducing pain. Although most studies reported that preoperative administration of NSAIDs was effective, some investigators found that NSAIDs administered alone were ineffective and no different from placebo.
De Geus et al. [31] (1990 to Sep/2016)	7/7	18–65 (min–max)	Ibuprofen vs other premedication drugs or placebo (oral route)	To evaluate the effects of ibuprofen compared to other drugs on the risk and intensity of postoperative pain resulting from endodontic treatment in adult patients	There is no clear evidence that preoperative ibuprofen is superior to other drugs in reducing the risk and intensity of post‐endodontic pain.
Iranmanesh et al. [32] (Jan/1966 to Jan/2017)	4/18	NR	Corticosteroids vs placebo or other control groups (any route)	To review the use of glucocorticosteroids in endodontics to assess the effect on pain following root canal treatment	It was not possible to combine the results of pretreatment corticosteroid use, as the route of administration may have influenced the outcome.
Jose et al. [33] (1983 to Apr/2020)	3/5	>18	NSAIDs and corticosteroids vs. placebo (oral route)	To compare the analgesic efficacy of NSAIDs and corticosteroids consumed through oral route for postoperative pain reduction.	Oral consumption of corticosteroids provides a better analgesic effect compared to NSAIDs. Although NSAIDs have a faster onset of action, corticosteroids produce a sustained analgesic effect over an extended period, resulting in superior pain relief. Oral premedication with corticosteroids also offers better anesthetic efficacy compared to NSAIDs.
Kumar et al. [34] (until Jun/2021)	10/10	14–65 (min–max)	NSAIDs and corticosteroids vs. placebo or active placebo (any route)	To assess the effectiveness of premedication in preventing post‐treatment pain following root canal treatment in patients with irreversible pulpitis.	The preoperative administration of anti‐inflammatory drugs effectively reduces postoperative pain for up to 24 h in teeth with irreversible pulpitis. This pain reduction may also help minimize the incidence of adverse effects associated with the repeated intake of postoperative analgesics. However, the low level of evidence using the GRADE approach, and the limited number of studies indicate that further trials are needed to validate this hypothesis.
Nagendrababu et al. [35] (until Jun/2017)	16/16	18–65 (min–max)	Oral premedication (alone or in combination) vs. other drugs (alone or in combination) or placebo or no treatment (oral route)	To identify the oral premedication that is most effective in reducing postoperative pain in adult patients after nonsurgical root canal treatment	Oral premedication can reduce postoperative pain after nonsurgical root canal treatment. Further research is needed on the use of narcotic agents for postoperative pain control in nonsurgical root canal procedures; piroxicam or prednisolone would be the preferred premedication options.
Nath et al. [36] (until Jan/2018)	6/14	18–67 (min–max)	Corticosteroids vs placebo or active placebo or no treatment (systemic or local administration)	To determine if the use of corticosteroids in endodontic treatment can reduce postoperative endodontic pain	Due to the high risk of bias and significant heterogeneity in clinical data, the authors call for more high‐quality, double‐blinded studies in the future to further elucidate the efficacy of corticosteroid use for pain reduction in endodontic treatment.
Santini et al. [37] (until Dec/2017)	4/10	16–65 (min–max)	Medications used for pain control vs. placebo or other medications to pain reduction (any route)	To evaluate the efficacy and safety of medications used to prevent and treat postoperative endodontic pain, to verify which therapy is the most indicated for clinical practice	There is an insufficient number of randomized controlled trials (RCTs) using consistent methodological standards to define systemic treatment protocols for managing postoperative endodontic pain.
Shamszadeh et al. [23] (until Oct/2017)	6/18	>15	Corticosteroids vs. placebo (any route)	To assess the efficacy of corticosteroids on postoperative endodontic pain	Pretreatment administration did not show a statistically significant difference at 6, 12, or 24 h.
Shirvani et al. [38] (until 2016)	6/27	>15	NSAIDs and/or paracetamol vs. placebo (oral and intraligamentary route)	To evaluate the efficacy of non‐narcotic analgesics including non‐steroidal anti‐inflammatory drugs (NSAIDs) and/or paracetamol in the treatment of postoperative endodontic pain	Pretreatment administration resulted in better analgesia at 12 h. The efficacy of various types of analgesics may be influenced by other treatment strategies.
Smith et al. [39] (until Dec/2015)	5/15	NR	NSAIDs (alone or in combination with other analgesics) vs. placebo or other non‐narcotic analgesics (oral route)	To evaluate the comparative efficacy of NSAIDs alone or in combination with other analgesics and other non‐narcotic drugs for postoperative endodontic discomfort in patients who present with pre‐treatment pain	There are insufficient data to recommend the most effective NSAID, dosage, or dosing interval for the relief of long‐duration postoperative endodontic pain in patients with preoperative pain.
Suneelkumar et al. [40] (until Feb/2018)	5/5	18–65 (min–max)	Corticosteroids vs. placebo or other control groups (any route)	To find the effect of preoperative use of corticosteroids on postendodontic pain in patients with symptomatic pulpitis who underwent root canal treatment in a single visit	The preoperative administration of single‐dose corticosteroids, such as prednisolone and dexamethasone, in symptomatic pulpitis can reduce the incidence of postoperative pain after single‐visit root canal therapy in the short term and decrease the need for rescue medication, with no reported drug‐related adverse effects.
Teja et al. [22] (Jan/1990 to May/2018)	6/6	18–65 (min–max)	NSAIDs vs. placebo or other non‐narcotic analgesics (oral route)	To evaluate the postoperative pain levels and analgesic intake on preoperative oral administration of non‐steroidal anti‐Inflammatory drugs in single visit root canal treatment.	Ibuprofen is considered the best drug of choice for single‐visit endodontics. It is also justified that pain after a single‐visit root canal treatment may last for 24 to 72 h, after which the patient typically experiences no pain.

Abbreviations: COX‐2, cyclooxygenase‐2; GRADE, Grading of Recommendations Assessment, Development and Evaluation; NR, not reported; NSAIDs, non‐steroidal anti‐inflammatory drugs; RCT, randomized controlled trial.

The patients in these studies ranged from 14 to 71 years, and six systematic reviews did not report the age of participants [23, 30, 32, 33, 38, 39].

Four studies evaluated the effect of NSAIDs [22, 31, 38, 39], although one study [31] assessed only ibuprofen. Corticosteroids were evaluated in four systematic reviews [23, 32, 36, 40]. Three studies [30, 33, 34] assessed the efficacy of NSAIDs and corticosteroids. One study assessed the effectiveness of NSAIDs, corticosteroids, and analgesic opioids [13]. Two systematic reviews examined the use of any drug for pain control [35, 37].

In general, the reviews included RCTs that used drugs through different routes, with the oral route being present in most studies.

### 3.2. Report of Pain Outcome for Quantitative Systematic Reviews

Six systematic reviews conducted meta‐analyses focusing on the pretreatment use of drugs to reduce postoperative endodontic pain. Five systematic reviews used conventional pairwise meta‐analysis [23, 31, 34, 36, 40], and one used network meta‐analysis [35]. The results of the meta‐analyses are shown in Table 2.

**Table 2:** Results of meta‐analysis of pretreatment corticosteroids (*n* = 4) and NSAIDs (*n* = 3).

References	Outcomes	Number of RCT (number of participants: intervention/control group)	Meta‐analysis results (95% CI) *I* ^2^	Author’s results
**CORTICOSTEROIDS (*n* = 4)**				

Kumar et al. [34]	Reduction in postoperative pain (VAS) (oral corticosteroid [prednisolone 30–40 mg] vs control) follow‐up: 6 h	RCT = 3 (226/218)	MD = −0.99 (−1.71 to −0.28) *I* ^2^ = 67%	Oral corticosteroids were superior to control GRADE = NR
	Reduction in postoperative pain (VAS) (oral corticosteroid [prednisolone 30–40 mg] vs control) follow‐up: 12 h	RCT = 3 (226/218)	MD = −1.19 (−1.95 to −0.44) *I* ^2^ = 68%	Oral corticosteroids were superior to control GRADE = NR
	Reduction in postoperative pain (VAS) (oral corticosteroid [prednisolone 30–40 mg] vs control) follow‐up: 24 h	RCT = 3 (226/218)	MD = −1.39 (−2.69 to −0.10) *I* ^2^ = 88%	Oral corticosteroids were superior to control GRADE = NR
	Reduction in postoperative pain (VAS or HP VAS) (parenteral corticosteroid [dexamethasone IL or submucosal 0.2 mL/4 mg/mL] vs control) follow‐up: 6 h	RCT = 2 (50/70)	MD = −1.31 (−2.18 to −0.44) *I* ^2^ = 78%	Parenteral corticosteroids were superior to control GRADE = NR
	Reduction in postoperative pain (VAS or HP VAS) (parenteral corticosteroid [dexamethasone IL or submucosal 0.2 mL/4 mg/mL] vs control) follow‐up: 12 h	RCT = 2 (50–70)	MD = −0.94 (−1.33 to −0.56) *I* ^2^ = 0%	Parenteral corticosteroids were superior to control GRADE = NR
	Reduction in postoperative pain (VAS or HP VAS) (parenteral corticosteroid [dexamethasone IL or submucosal 0.2 mL/4 mg/mL] vs control) follow‐up: 24 h	RCT = 2 (50/70)	MD = −0.71 (−1.02 to −0.43) *I* ^2^ = 38%	Parenteral corticosteroids were superior to control GRADE = NR

Nagendrababu et al. [35]	Reduction in postoperative pain (VAS) (prednisolone 30–40 mg oral route vs placebo) follow‐up: 6 h	RCT = 3 (251/250)	MD = −20.18 (−36.54 to −3.82) *I* ^2^ = 91.3%	Prednisolone was superior to placebo GRADE = VERY LOW
	Reduction in postoperative pain (VAS) (prednisolone 30–40 mg oral route vs placebo) follow‐up: 12 h	RCT = 3 (251/250)	MD = −20.43 (−28.22 to −12.64) *I* ^2^ = 65.6%	Prednisolone was superior to placebo GRADE = MODERATE
	Reduction in postoperative pain (VAS) (prednisolone 30–40 mg oral route vs placebo) follow‐up: 24 h	RCT = 3 (251/250)	MD = −21.23 (−36.42 to −6.04) *I* ^2^ = 92.6%	Prednisolone was superior to placebo GRADE = VERY LOW

Nath et al. [36]	Reduction in postoperative pain (VAS) (corticosteroids [prednisolone oral route 30–40 mg; dexamethasone oral route 4 mg or IL 0.2 mg] vs placebo) follow‐up: 4–6 h	RCT = 5 (295/290)	MD = −21.15 (−35.03 to −7.26) *I* ^2^ = NR	Corticosteroids were superior to placebo GRADE = LOW
	Reduction in postoperative pain (VAS) (corticosteroids vs placebo) follow‐up: 12 h	RCT = NR (NR/NR)	MD = −19.57 (−34.77 to −4.37) *I* ^2^ = NR	Corticosteroids were superior to placebo GRADE = NR
	Reduction in postoperative pain (VAS) (corticosteroids [prednisolone oral route 30–40 mg; dexamethasone oral route 4 mg or IL 0.2 mg; methylprednisolone IL 4–8 mg] vs placebo) follow‐up: 24 h	RCT = 6 (313/300)	MD = −17.38 (−39.07 to 4.31) *I* ^2^ = NR	Corticosteroids were not significantly superior to placebo GRADE = LOW
	Reduction in postoperative pain (VAS)—subgroup analysis (IL injection corticosteroids [methylprednisolone 4–8 mg; dexamethasone 0.2 mg] vs placebo) follow‐up: 4–24 h	RCT = 2 (38/30)	MD = −25.17 (−34.87 to −15.46) *I* ^2^ = 0%	Intraligamental corticosteroids were superior to placebo GRADE = NR
	Reduction in postoperative pain (VAS)—subgroup analysis (Oral corticosteroids [prednisolone oral route 30–40 mg; dexamethasone oral route 4 mg] vs placebo) follow‐up: 12 h	RCT = 4 (275/270)	MD = −19.63 (−37.07 to −2.19) *I* ^2^ = 98%	Oral corticosteroids were superior to placebo GRADE = NR
	Reduction in postoperative pain (VAS)—subgroup analysis (dexamethasone oral route 4 mg or IL injection 0.2 mL] vs placebo) follow‐up: 4–6	RCT = 2 (45/43)	MD = −18.09 (−39.99 to 3.81) *I* ^2^ = NR	Dexamethasone was not significantly superior to placebo GRADE = NR
	Reduction in postoperative pain (VAS)—subgroup analysis (prednisolone 30–40 mg oral route vs placebo) follow‐up: 4–6 h	RCT = 3 (250/247)	MD = −23.07 (−40.37 to −5.76) *I* ^2^ = NR	Prednisolone was superior to placebo GRADE = NR

Suneelkumar et al. [40]	Reduction in postoperative pain (VAS) (prednisolone 30–40 mg oral route vs placebo) follow‐up: 6 h	RCT = 3 (226/225)	MD = −21.93 (−35.72 to −8.13) *I* ^2^ = 80%	Prednisolone was superior to placebo GRADE = VERY LOW
	Reduction in postoperative pain (VAS) (prednisolone 30–40 mg oral route vs placebo) follow‐up: 12 h	RCT = 3 (226/225)	MD = −22.21 (−28.69 to −15.72) *I* ^2^ = 31%	Prednisolone was superior to placebo GRADE = MODERATE
	Reduction in postoperative pain (VAS) (prednisolone 30–40 mg oral route vs placebo) follow‐up: 24 h	RCT = 3 (226/225)	MD = −22.57 (−37.14 to −8.00) *I* ^2^ = 87%	Prednisolone was superior to placebo GRADE = VERY LOW

**NSAIDs (*n* = 3)**				

De Geus et al. [31]	Pain intensity (VAS, NRS) (Ibuprofen 200, 400, or 600 mg vs other drugs [tenoxicam 20 mg, rofecoxib 50 mg, indomethacin 25 mg, and zintoma 2 g]) follow‐up: 6–8 h	RCT = 5 (116/116)	SMD = −0.24 (−0.65 to 0.16) *I* ^2^ = 57%	Ibuprofen was not superior to other compared drugs GRADE = LOW
	Pain intensity (VAS or NRS) (Ibuprofen 200, 400, or 600 mg vs other drugs [tenoxicam 20 mg, rofecoxib 50 mg, indomethacin 25 mg, and zintoma 2 g]) follow‐up: 24 h	RCT = 5 (116/116)	SMD = −0.01 (−0.40 to 0.39) *I* ^2^ = 55%	Ibuprofen was not superior to other compared drugs GRADE = LOW
	Pain intensity (VAS or NRS) (Ibuprofen 200, 400, or 600 mg vs placebo) follow‐up: 6–8 h	RCT = 6 (130/128)	SMD = −0.72 (−1.53 to 0.09) *I* ^2^ = 89%	Ibuprofen was not superior to placebo GRADE = VERY LOW
	Pain intensity (VAS or NRS) (Ibuprofen 200, 400, or 600 mg vs placebo) follow‐up: 24 h	RCT = 6 (130/128)	SMD = −0.35 (−0.96 to 0.26) *I* ^2^ = 81%	Ibuprofen was not superior to placebo GRADE = VERY LOW
	Pain intensity (VAS or NRS)—sensitivity analysis (Ibuprofen 200, 400 or 600 mg vs placebo) follow‐up: 24 h	RCT = 5 (116/116)	SMD = −0.61 (−1.05 to −0.17) *I* ^2^ = 61%	Ibuprofen was superior to placebo in sensitivity analysis GRADE = LOW

Nagendrababu et al. [35]	Reduction in postoperative pain (VAS) (NSAIDs [ibuprofen 400 or 600 mg; diclofenac 100 mg; sulindac 200 mg; ketorolac 20 mg] vs placebo) follow‐up: 6 h	RCT = 4 (107/91)	MD = −5.75 (−18.01 to 6.51) *I* ^2^ = 91.6%	NSAIDs were not significantly superior to placebo GRADE = VERY LOW
	Reduction in postoperative pain (VAS) (NSAIDs [ibuprofen 400 or 600 mg; sulindac 200 mg; ketorolac 20 mg; piroxicam 40 mg; lornoxicam 8 mg] vs placebo) follow‐up: 12 h	RCT = 6 (173/135)	MD = −7.20 (−18.06 to 3.66) *I* ^2^ = 97.6%	NSAIDs were not significantly superior to placebo GRADE = VERY LOW
	Reduction in postoperative pain (VAS) (NSAIDs [ibuprofen 400 or 600 mg; sulindac 200 mg; ketorolac 20 mg; piroxicam 40 mg; lornoxicam 8 mg] vs placebo) follow‐up: 24 h	RCT = 6 (173/135)	MD = −3.55 (−11.91 to 4.81) *I* ^2^ = 95.3%	NSAIDs were not significantly superior to placebo GRADE = VERY LOW
	Pain intensity (VAS) (ibuprofen 400 or 600 mg oral route vs placebo) follow‐up: 6 h	RCT = 3 (76/61)	MD = −5.56 (−22.69 to 11.57) *I* ^2^ = 93.5%	Ibuprofen was not superior to placebo GRADE = NR
	Pain intensity (VAS) (ibuprofen 400 or 600 mg oral route vs placebo) follow‐up: 12 h	RCT = 3 (68/53)	MD = 2.48 (−3.02 to 7.98) *I* ^2^ = 73.5%	Ibuprofen was not superior to placebo GRADE = NR
	Pain intensity (VAS) (ibuprofen 400 or 600 mg oral route vs placebo) follow‐up: 24 h	RCT = 3 (68/53)	MD = 2.20 (−6.11 to 10.51) *I* ^2^ = 92.8%	Ibuprofen was not superior to placebo GRADE = NR

Kumar et al. [34]	Reduction in postoperative pain (VAS or HP VAS) (oral NSAIDs [diclofenac 50 mg or ketorolac 30 mg] vs control) follow‐up: 6 h	RCT = 2 (48/41)	MD = −0.48 (−0.91 to −0.05) *I* ^2^ = 0%	Oral NSAIDs were superior to control GRADE = NR
	Reduction in postoperative pain (VAS or HP VAS) (oral NSAIDs [diclofenac 50 mg or ketorolac 30 mg] vs control) follow‐up: 12 h	RCT = 2 (48/41)	MD = −0.36 (−0.79 to 0.06) *I* ^2^ = 0%	Oral NSAIDs were not significantly superior to control GRADE = NR
	Reduction in postoperative pain (VAS or HP VAS) (oral NSAIDs [diclofenac 50 mg or ketorolac 30 mg] vs control) follow‐up: 24 h	RCT = 2 (48/41)	MD = −0.35 (−0.88 to 0.18) *I* ^2^ = 0%	Oral NSAIDs were not significantly superior to control GRADE = NR
	Reduction in postoperative pain (VAS or HP VAS) (parenteral NSAIDs [piroxicam IL 0.4 mL of 20 mg/mL or ketorolac 30 mg/mL] vs control) follow‐up: 24 h	RCT = 2 (65/60)	MD = −0.65 (−1.01 to −0.29) *I* ^2^ = 0%	Parenteral NSAIDs were superior to control GRADE = NR

Abbreviations: CI, confidence interval; GRADE, Grading of Recommendations Assessment, Development and Evaluation; HP‐VAS, Heft Parker visual analog scale; *I* ^2^, heterogeneity; MD, mean difference; NR, not reported; NRS, Numerical Rating Scale: none, mild, moderate, and severe pain; NSAIDs, non‐steroidal anti‐inflammatory drugs; RCT, randomized controlled trials; SMD, standardized mean difference; VAS, visual analog scale.

The results of Shamszadeh et al. [23] are not reported in Table 2 because the primary studies mentioned were also included in Nagendrababu et al. [35]. Meta‐analyses including only one RCT are not included in Table 2 and discussed based on the results of primary studies (Tables 3 and 4).

**Table 3:** Randomized controlled clinical trials included in systematic reviews of pretreatment corticosteroids (*n* = 11).

RCT (number of SR)	P: participants (age in years) I: intervention (n) C: control (n) O: outcomes (scale) follow‐up	Results (*p*‐Value)
**DEXAMETHASONE (*n* = 7)**		

Aksoy and Ege [8] (1)	P: 18–65 years I1: dexamethasone 8 mg/2 mL submucosal injection (*n* = 30) I2: tramadol 100 mg/2 mL submucosal injection (*n* = 30) C: placebo (*n* = 30) O: reduction in pain (HP VAS) Follow‐up: 6, 12, 24, 48, and 72 h	Dexamethasone was superior to placebo at 6, 12, 24, and 48 h (*p*‐value < 0.001). Dexamethasone was superior to tramadol at 6 and 12 h (*p*‐value < 0.0167). No significant variance was observed between the dexamethasone and tramadol groups at 24, 48, and 72 h (*p*‐value > 0.0167). At 72 h, no significant difference was found between the experimental groups and the control group (*p*‐value > 0.0167).
Jorge‐Araújo et al. [10] (2)	P: 18–66 years I1: dexamethasone 8 mg oral (*n* = 20) I2: ibuprofen 400 mg oral (*n* = 20) C: placebo (*n* = 20) O: reduction in pain (NRS) Follow‐up: 4, 8, 12, 24, and 48 h	There was no significant difference among groups (*p*‐value > 0.05).
Konagala et al. [20] (2)	P: 18–50 years I1: dexamethasone 4 mg oral (*n* = 33) I2: piroxicam 20 mg (*n* = 33) I3: deflazacort 30 mg (*n* = 33) C: placebo (*n* = 33) O: reduction in pain (VAS) Follow‐up: 6, 12, 24, 48, 7and 2 h	Dexamethasone or piroxicam or deflazacort was superior to placebo at 6, 12, and 24 h (*p*‐value < 0.05). There was no significant difference among groups at 48 and 72 h (*p*‐value > 0.05).
Mehrvarzfar et al. [41] (6)	P: 18–65 years I: 0.2 mL of dexamethasone 8 mg/2mL IL (*n* = 20) C1: passive placebo (*n* = 20) C2: active placebo (2% lidocaine containing 1:80000 epinephrine) (*n* = 20) O: reduction in pain (VAS) Follow‐up: 6, 12, 24, 48, and h	Dexamethasone was superior to placebo at 6, 12, and 24 h. Pain at 6 h: *p*‐value < 0.01 Pain at 12 h: *p*‐value < 0.001 Pain at 24 h: *p*‐value < 0.01 Pain at 48 h: *p*‐value < 0.8
Mello [42] (1)	P: 18–60 years I1: dexamethasone 4 mg oral (*n* = 33) I2: ibuprofen 600 mg (*n* = 33) C: placebo (*n* = 33) O: reduction in pain (VAS) Follow‐up: 4, 6, 2, and 4 h	Dexamethasone or ibuprofen promoted a significant reduction of the post‐operative pain after 4 or 6 h. No statistically significant difference was found among dexamethasone and ibuprofen after 4 or 6 h and among the three groups after 24 h. Pain at 4 h: *p*‐Value = 0.01 (dexamethasone vs. placebo) *p*‐Value > 0.05 (dexamethasone vs. ibuprofen) Pain at 6 h: *p*‐Value < 0.0001 (dexamethasone vs. placebo) *p*‐Value > 0.05 (dexamethasone vs. ibuprofen) Pain at 24 h: *p*‐value > 0.05 (dexamethasone vs. placebo) *p*‐value > 0.05 (dexamethasone vs. ibuprofen)
Pochapski et al. [19] (5)	P: 18–67 years (mean: 42.1 years) I: dexamethasone 4 mg oral (*n* = 25) C: placebo (*n* = 25) O: reduction in pain (NRS) Follow‐up: 4, 12, 24, 4, and 8 h	Dexamethasone was superior to placebo at 4 and 12 h (*p*‐value < 0.05). There was no significant difference between groups at 24 and 48 h (*p*‐value > 0.05).
Sharma et al. [43] (3)	P: 18–50 years I1: dexamethasone 4 mg oral (*n* = 20). I2: dexamethasone 4 mg/mL IM (*n* = 20) I3: dexamethasone 4 mg/mL IL (*n* = 20) I4: dexamethasone 4 mg/mL supraperiosteal (*n* = 20) C: placebo (*n* = 20) O: reduction in pain (VAS) Follow‐up: 6, 12, 2, and 4 h	Oral and supraperiosteal dexamethasone was superior to placebo at 6, 12, and 24 h (*p*‐value < 0.05)–mean percentage pain reduction (MPPR) Oral dexamethasone was superior to dexamethasone IL at 6, 12, and 24 h (*p*‐value < 0.05) Oral dexamethasone was superior to dexamethasone IM and supraperiosteal at 6 h (*p*‐value < 0.05) Oral administration of dexamethasone resulted in significantly more percentage of reduction in preoperative pain as compared to other routes after 24 h (*p*‐value < 0.05).

**PREDNISOLONE (*n* = 3)**		

Elkhadem et al. [7] (5)	P: 18–35 years I: prednisolone 40 mg (2 × 20 mg) Oral (*n* = 200) C: placebo (*n* = 200) O: reduction in pain (VAS) Follow‐up: 6, 12, 2, and 4 h	Prednisolone was superior to placebo at 6, 12, and 24 h (*p* < 0.001). Pain at 6 h: RRR: 20.31% (12.03% to 27.82%) Pain at 12 h: RRR: 23.39% (14.75% to 31.16%) Pain at 24 h: RRR: 28.85% (18.08% to 38.20%) NNT (24 h) = 4
Jalalzadeh et al. [44] (6)	P: 18–59 years I: prednisolone 30 mg oral (*n* = 29) C: placebo (*n* = 34) O: reduction in pain (VAS) Follow‐up: 6, 12, 2, and 4 h	Prednisolone was superior to placebo at 6, 12, and 24 h. Pain at 6 h: *p*‐value < 0.05 Pain at 12 h: *p*‐value < 0.05 Pain at 24 h: *p*‐value < 0.05
Praveen et al. [11] (7)	P: 18–50 years I1: prednisolone 30 mg oral (*n* = 30) I2: ketorolac 20 mg oral (*n* = 29) C: placebo (*n* = 27) O: reduction in pain (VAS) Follow‐up: immediately after treatment completion, 6, 12, 24, 4, and 8 h	Prednisolone was superior to placebo and ketorolac at 12, 24, and 48 h. Ketorolac was superior to placebo and prednisolone at 6 h. No statistically significant difference was found between prednisolone and placebo at 6 h. Pain at 6 h: *p*‐value = 0.044 (3 groups) Pain at 12 h: *p*‐value = 0.001 (3 groups) Pain at 24 h: *p*‐value = 0.001 (3 groups) Pain at 48 h *p*‐value = 0.001 (3 groups)

**DEFLAZACORT (*n* = 1)**		

Konagala et al. [20] (2)	P: 18–50 years I1: deflazacort 30 mg oral (*n* = 33) I2: piroxicam 20 mg oral (*n* = 33) I3: dexamethasone 4 mg oral (*n* = 33) C: placebo (*n* = 33) O: reduction in pain (VAS) Follow‐up: 6, 12, 24, 48, 7, and 2 h	Deflazacort or dexamethasone or piroxicam was superior to placebo at 6, 12, and 24 h (*p*‐value < 0.05). There was no significant difference among groups at 48 and 72 h (*p*‐value > 0.05).

**METHYLPREDNISOLONE (*n* = 1)**		

Kaufman et al. [45] (4)	P: 19–71 years I: slow‐release methylprednisolone 4–8 mg IL (*n* = 18)C1: placebo (anesthesia as passive control) (*n* = 10) C2: 3% mepivacaine (*n* = 17) O: reduction in pain (0–10 scale) Follow‐up: 24 h	Methylprednisolone IL was superior to placebo within 24 h (*p*‐value = 0.0228).

Abbreviations: IL, intraligamentary; IM, intramuscular; NNT, number needed to treat; NRS, numeric rating scale; RCT, randomized controlled trial; RRR, relative risk reduction; SR, systematic review; VAS, visual analog scale.

**Table 4:** Randomized controlled trials included in systematic reviews of pretreatment NSAIDs (*n* = 28).

RCT (number of SR)	P: Participants (age in years) I: intervention (n) C: control (n) O: outcomes (scale) Follow‐up	Results (*p*‐value)
**IBUPROFEN (*n* = 14)**		

Arslan et al. [46] (7)	P: 18–52 years I1: ibuprofen 200 mg Oral (*n* = 16) I2: tenoxicam 20 mg Oral (*n* = 16) C: placebo (*n* = 16) O: reduction in pain (VAS) Follow‐up: 6, 12, 24, 48, and 72 h	Tenoxicam or ibuprofen was superior to placebo at 6 h (*p*‐value = 0.000). There was no significant difference between tenoxicam and ibuprofen at 6 h (*p*‐value = 0.723). There was no significant difference among groups at 12, 24, 48, and 72 h (*p*‐value > 0.05).
Attar et al. [16] (7)	P: Ibuprofen tablet (44.9 ± 4.0 years); Ibuprofen liquigel (41.6 ± 4.3 years); and placebo (45.8 ± 5.1 years) I1: ibuprofen tablet 600 mg oral (*n* = 14) I2: ibuprofen liquigel 600 mg oral (*n* = 13) C: placebo (*n* = 12) O: reduction in pain (VAS) Follow‐up: 6, 12, 18, and 24 h	There was no significant difference among groups at 6, 12, 18, and 24 h (*p*‐value *=* 0.84).
Bali et al. [15] (1)	P: not obtained I1: ibuprofen 200 mg oral (*n* = 30) I2: tenoxicam 20 mg oral (*n* = 30) C: ketorolac 10 mg oral (*n* = 30) O: reduction in pain (VAS) Follow‐up: 6, 12, 24, 48, and 72 h	There was no significant difference among the three analgesics at 6 h (*p*‐value = 0.723). There was no significant difference between ibuprofen and ketorolac at 12, 24, 48, and 72 h (*p*‐value > 0.05).
Dejkam et al. [47] (1)	P: 18–65 years I1: ibuprofen 400 mg oral (Gelofen) (*n* = 20) I2: ibuprofen 200 mg + acetaminofen 325 mg + caffeine 40 mg oral (Novafen) (*n* = 20) C: placebo (*n* = 20) O: reduction in pain (VAS) Follow‐up: 4, 8, 12, 24, and 48 h	Novafen was superior to Gelofen and placebo at 8 h. There was no significant difference between groups at 4, 12, 24, and 48 h. Pain at 4 h: *p*‐value = 0.16 Pain at 8 h: *p*‐value = 0.03 Pain at 12 h: *p*‐value = 0.18 Pain at 24 h: *p*‐value = 0.95 Pain at 48 h *p*‐value = 0.59
Ehsani et al. [48] (1)	P: NR I1: NAC 400 mg oral (*n* = 20) I2: ibuprofen 400 mg oral (*n* = 20) I3: NAC 400 mg/ibuprofen 20 mg oral (*n* = 20) C: placebo (*n* = 20) O: measurement of TNF‐α, IL‐6, and IL‐17 levels Reduction in pain (VAS) Follow‐up: 4, 8, 12, and 24 h	Ibuprofen was superior to placebo at 4 h (*p*‐value = 0.017). NAC group was superior to placebo at 8 h (*p*‐value = 0.033 and 12 h (*p*‐value = 0.049). There was no significant difference among groups at 24 h (*p*‐value > 0.05).
Gopikrishna and Parameswaran [14] (6)	P: 18–65 years I1: rofecoxib 50 mg oral (*n* = 15) I2: ibuprofen 600 mg oral (*n* = 15) C: placebo (*n* = 15) O: reduction in pain (VAS) Follow‐up: 4, 8, 12, 24, 48, and 72 h	Ibuprofen or rofecoxib was superior to placebo at 4 and 8 h (*p*‐value < 0.05). There was no significant difference between rofecoxib and ibuprofen during both 4 h (*p*‐value = 0.30) and 8 h (*p*‐value = 0.25) periods. Rofecoxib was superior to ibuprofen and placebo at 12 h (*p*‐value = 0.003) and 24 h (*p*‐value = 0.047).
Khorasani and Mahmudi [49] (2)	P: 25–50 years I1: Ibuprofen 400 mg oral (*n* = 16) I2: sulindac 200 mg oral (*n* = 16) C: placebo (*n* = 16) O: reduction in pain (VAS) Follow‐up: 6, 12, 24, 48, and 72 h	There was no significant difference between groups at 6, 12, 24, 48, and 72 h (*p*‐value > 0.05).
Jorge‐Araújo et al. [10] (2)	P: 18–66 years I1: dexamethasone 8 mg oral (*n* = 20) I2: ibuprofen 400 mg oral (*n* = 20) C: placebo (*n* = 20) O: reduction in pain (NRS) Follow‐up: 4, 8, 12, 24, and 48 h	There was no significant difference among groups at 4, 8, 12, 24 and 48 h (*p*‐value > 0.05).
Mello [42] (1)	P: 18–60 years I1: dexamethasone 4 mg oral (*n* = 33) I2: ibuprofen 600 mg oral (*n* = 33) C: placebo (*n* = 33) O: reduction in pain (VAS) Follow‐up: 4, 6, and 24 h	Ibuprofen or dexamethasone was superior to placebo after 4 and 6 h. No statistically significant difference was found among the three groups after 24 h. Pain at 4 h: *p*‐value = 0.0057 (ibuprofen vs. placebo) *p*‐Value > 0.05 (ibuprofen vs. dexamethasone) Pain at 6 h: *p*‐value = 0.0063 (ibuprofen vs. placebo) *p*‐Value > 0.05 (ibuprofen vs. dexamethasone) Pain at 24 h: *p*‐value > 0.05 (ibuprofen vs. placebo) *p*‐Value > 0.05 (ibuprofen vs. dexamethasone)
Menke et al. [50] (5)	P: 18 years I1: etodolac 400 mg oral (*n* = 12) I2: ibuprofen 600 mg oral (*n* = 12) C1: placebo (*n* = 12) O: reduction in pain (VAS) Follow‐up: 4, 8, 12, 24, 48, and 72 h	Ibuprofen was superior to etodolac or placebo at 4 h (*p*‐value = 0.0111) and 8 h (*p*‐value = 0.0397). There was no significant difference between ibuprofen and placebo at 12, 24, 48, and 72 h (*p*‐value > 0.05).
Mirzaie et al. [51] (2)	P: 18–65 years I1: celecoxib 200 mg oral (*n* = 30) I2: ibuprofen 400 mg oral (Gelofen) (*n* = 30) C: placebo (*n* = 30) O: reduction in pain (VAS) Follow‐up: 4, 8, 12, and 24 h	Ibuprofen or celecoxib was superior to placebo at 8 and 12 h (*p*‐value < 0.05). There was no significant difference among the three groups at 4 and 24 h (*p*‐value > 0.05).
Mokhtari et al. [52] (6)	P: 19–30 years I1: ibuprofen 400 mg oral (*n* = 22) I2: indomethacin 25 mg oral (*n* = 22) C1: placebo (*n* = 22) O: reduction in pain (VAS) Follow‐up: 8, 12, and 24 h	Ibuprofen or indomethacin was superior to placebo at 8 h (*p*‐value < 0.001 and *p*‐value = 0.001, respectively). There was no significant difference among the three groups at 12 h (*p*‐value = 0.80) and 24 h (*p*‐value = 0.27).
Ramazani et al. [53] (4)	P: 18–65 years I1: ibuprofen 400 mg oral (*n* = 30) I2: zintoma 10 mg oral (*n* = 30) C: placebo (*n* = 30) O: reduction in pain (VAS) Follow‐up: 4, 8, 12, 24, 48, and 72 h	Ibuprofen was superior to zintoma or placebo at all times (*p*‐value < 0.05).
Saatchi et al. [54] (3)	P: not obtained I1: ibuprofen 400 mg oral (*n* = 30) I2: slow‐released diclofenac sodium 100 mg oral (*n* = 30) C: placebo (*n* = 30) O: reduction in pain (VAS) Follow‐up: 2, 6, 10, 18, 36, 44, 54, 66, and 72 h	Ibuprofen was superior to slow‐released diclofenac sodium in the first 2 h (*p*‐value = 0.01). Slow‐released diclofenac sodium was superior to ibuprofen at 10, 18, and 36 h (*p*‐value < 0.001).

**KETOROLAC (*n* = 6)**		

Akhlaghi et al. [55] (1)	P: 18–65 years I: ketorolac 30 mg in 1 mL IL (*n* = 34) C: placebo (*n* = 34) O: reduction in pain (HP‐VAS) Follow‐up: 2, 4, 6, and 24 h	Ketorolac IL was superior to placebo in overall evaluations (*p*‐value < 0.0001) and in each time interval (*p*‐value = 0.043).
Bali et al. [15] (1)	P: not obtained I1: ibuprofen 200 mg oral (*n* = 30) I2: tenoxicam 20 mg oral (*n* = 30) I3: ketorolac 10 mg oral (*n* = 30) O: reduction in pain (VAS) Follow‐up: 6, 12, 24, 48, and 72 h	There was no significant difference among the three analgesics at 6 h (*p* = 0.723). There was no significant difference between ibuprofen and the ketorolac at 12, 24, 48, and 72 h (*p* > 0.05).
Mellor et al. [56] (1)	P: 18–65 years I: ketorolac 30 mg in 1 mL IL (*n* = 5) C: placebo (*n* = 5) O: reduction in pain (VAS) Follow‐up: 6 and 24 h	Ketorolac IL was not superior to placebo (*p*‐value > 0.05).
Penniston and Hargreaves [57] (1)	P: 18–65 years I1: ketorolac 30 mg IM (*n* = 10) I2: ketorolac 30 mg periapical infiltration (*n* = 18) I3: 2% mepivacaine with 1:20,000 levonordefrin periapical (*n* = 10) C: placebo (*n* = 14) O: reduction in pain (4‐point category pain scale: none, mild, moderate, and severe; VAS; Heft‐Parker scale) Follow‐up: 3, 4, 5, and 6 h	Ketorolac periapical infiltration was superior to placebo (*p* < 0.05).
Praveen et al. [11] (7)	P: 18–50 years I1: prednisolone 30 mg oral (*n* = 30) I2: ketorolac 20 mg oral (*n* = 29) C: placebo (*n* = 27) O: reduction in pain (VAS) Follow‐up: immediately after treatment completion, 6, 12, 24, and 48 h	Ketorolac was superior to placebo and prednisolone at 6 h. Prednisolone was superior to placebo and ketorolac at 12, 24, and 48 h. Pain at 6 h: *p*‐Value = 0.044 (3 groups) Pain at 12 h: *p*‐Value = 0.001 (3 groups) Pain at 24 h: *p*‐Value = 0.001 (3 groups) Pain at 48 h *p*‐Value = 0.001 (3 groups)
Sethi et al. [58] (3)	P: 18–60 years I1: tapentadol 100 mg oral (*n* = 20) I2: etodolac 400 mg oral (*n* = 20) I3: ketorolac 10 mg oral (*n* = 20) O: reduction in pain (VAS) Follow‐up: 6, 12, 18, and 24 h	Ketorolac was superior to etodolac at 6, 12, 18, and 24 h (*p*‐value < 0.05). Ketorolac was superior to tapentadol at 6, 12, and 18 h (*p*‐value < 0.05). No significant difference was found between ketorolac and tapentadol at 24 h (*p*‐value > 0.05)

**DICLOFENAC (*n* = 5)**		

Al‐Rawhani et al. [9] (1)	P: 18–55 years (diclofenac group); 25–60 (placebo group) I: diclofenac potassium 50 mg Oral (*n* = 35) C: placebo (*n* = 35) O: reduction in pain (HP‐VAS) Follow‐up: 6, 12, 24, and 48 h	Diclofenac potassium was superior to placebo at 48 h. Pain at 6 h: *p*‐value = 0.301 Pain at 12 h: *p*‐value = 0.290 Pain at 24 h: *p*‐value = 0.815 Pain at 48 h: *p*‐value = 0.028
Jenarthanan and Subbarao [59] (1)	P: 18–65 years I1: diclofenac sodium 75 mg oral (*n* = 10) I2: diclofenac sodium IL injection (dose NR) (*n* = 10) C: placebo (Vitamin B_12_ tablets) (*n* = 10) O: reduction in pain (VAS) Follow‐up: 6, 12, 24, and 48 h	Diclofenac sodium was superior to placebo at 6, 12, 24, and 48 h. Pain at 6 h: *p*‐value < 0.001 Pain at 12 h: *p*‐value < 0.001 Pain at 24 h: *p*‐value < 0.001 Pain at 48 h: *p*‐value = 0.006 Intraligamentary route was superior to the oral route up to 48 h.
Metri et al. [60] (1)	P: 20–60 years I: diclofenac sodium 100 mg oral (*n* = 25) C: placebo (*n* = 25) O: reduction in pain (VAS) Follow‐up: 6, 12, and 24 h	Diclofenac sodium was superior to placebo at 6 and 12 h (*p* < 0.05). There was no significant difference at 24 h (*p* > 0.05).
Rashka et al. [61] (2)	P: not obtained I: diclofenac sodium 0.4 mL (30 mg) IL (*n* = 26) C: placebo lidocaine (*n* = 26) O: Reduction in pain (VAS) Follow‐up: 4, 8, 12, 24, and 48 h	Diclofenac sodium was superior to placebo at 4, 8, 12, 24, and 48 h.
Saatchi et al. [54] (3)	P: not obtained I1: ibuprofen 400 mg oral (*n* = 30) I2: slow‐released diclofenac sodium 100 mg oral (*n* = 30) C: placebo (*n* = 30) O: reduction in pain (VAS) Follow‐up: 2, 6, 10, 18, 36, 44, 54, 66, and 72 h	Slow‐released diclofenac sodium was superior to ibuprofen at 10, 18, and 36 h (*p* < 0.001). Slow‐released diclofenac sodium was superior to placebo at 2, 10, 18, and 36 h (*p* < 0.05). Ibuprofen was superior to slow‐released diclofenac sodium in the first 2 h (*p* = 0.01).

**PIROXICAM (*n* = 3)**		

Atbaei and Mortazavi [62] (3)	P: 14–65 years I: piroxicam (0.4 mL of 20 mg/mL) IL (*n* = 35) C: active placebo lidocaine 2% (*n* = 30) O: reduction in pain (VAS) Follow‐up: 4, 8, 12, 24, and 48 h	Piroxicam IL was superior to placebo at 4, 8, 12, 24, and 48 h. Pain at 4 h: *p*‐value = 0.025 Pain at 8 h: *p*‐value = 0.0001 Pain at 12 h: *p*‐value = 0.002 Pain at 24 h: *p*‐value = 0.011 Pain at 48 h: *p*‐value = 0.031
Joshi et al. [63] (2)	P: 18–65 years I1: piroxicam 40 mg oral (*n* = 22) I2: piroxicam (0.4 mL of 20 mg/mL) IL (*n* = 22) C: control (1.8 mL of 2% lidocaine containing 1:80,000 epinephrine) (*n* = 22) O: reduction in pain (VAS) Follow‐up: 4, 8, 12, 24, and 48 h	Piroxicam Oral or IL were superior to placebo at all times (*p* < 0.05). There was no significant difference between piroxicam oral and IL at 4 and 8 h (*p* < 0.448). Piroxicam IL was superior to oral route at 12, 24 and 48 h (*p* < 0.001).
Konagala et al. [20] (2)	P: 18–50 years I1: dexamethasone 4 mg oral (*n* = 33) I2: piroxicam 20 mg oral (*n* = 33) I3: deflazacort 30 mg oral (*n* = 33) C: placebo (*n* = 33) O: reduction in pain (VAS) Follow‐up: 6, 12, 24, 48, and 72 h	Piroxicam or dexamethasone or deflazacort was superior to placebo at 6, 12, and 24 h (*p*‐value < 0.05). There was no significant difference among groups at 48 and 72 h (*p*‐value > 0.05).

**CELECOXIB (*n* = 2)**		

Ashraf et al. [64] (3)	P: 18–57 years I: celecoxib 400 mg oral (*n* = 15) C: placebo (*n* = 15) O: reduction in pain (Heft‐Parker VAS) Follow‐up: 4, 8, 12, 24, and 48 h	There was no significant difference among groups at 4, 8, 12, 24, and 48 h (*p*‐value > 0.05).
Mirzaie et al. [51] (2)	P: 18–65 years I1: celecoxib 200 mg oral (*n* = 30) I2: ibuprofen 400 mg oral (Gelofen) (*n* = 30) C: placebo (*n* = 30) O: reduction in pain (VAS) Follow‐up: 4, 8, 12, and 24 h	Celecoxib or ibuprofen was superior to placebo at 8 and 12 h (*p*‐value < 0.05). There was no significant difference among three groups at 4 and 24 h (*p* > 0.05).

**ETODOLAC (*n* = 2)**		

Menke et al. [50] (5)	P: 18 year old I1: etodolac 400 mg oral (*n* = 12) I2: ibuprofen 600 mg oral (*n* = 12) C1: placebo (*n* = 12) O: reduction in pain (VAS) Follow‐up: 4, 8, 12, 24, 48, and 72 h	Ibuprofen was superior to etodolac or placebo at 4 h (*p*‐value = 0.0111) and 8 h (*p*‐value = 0.0397).
Sethi et al. [58] (3)	P: 18–60 years I1: tapentadol 100 mg oral (*n* = 20) I2: etodolac 400 mg oral (*n* = 20) I3: ketorolac 10 mg oral (*n* = 20) O: reduction in pain (VAS) Follow‐up: 6, 12, 18, and 24 h	Ketorolac was superior to etodolac at 6, 12, 18, and 24 h (*p* < 0.05).

**OTHER NSAIDs (*n* = 7)**		

Bali et al. [15] (1)	P: not obtained I1: ibuprofen 200 mg oral (*n* = 30) I2: tenoxicam 20 mg oral (*n* = 30) C: ketorolac 10 mg oral (*n* = 30) O: reduction in pain (VAS) Follow‐up: 6, 12, 24, 48, and 72 h	There was no significant difference between the three analgesics at 6 h (*p*‐value = 0.723). There were no significant ibuprofen and the ketorolac at 12, 24, 48, and 72 h (*p*‐value > 0.05).
Dejkam et al. [47] (1)	P: 18–65 years I1: ibuprofen 400 mg oral (Gelofen) (*n* = 20) I2: ibuprofen 200 mg + acetaminofen 325 mg + caffeine 40 mg oral (Novafen) (*n* = 20) C: placebo (*n* = 20) O: reduction in pain (VAS) Follow‐up: 4, 8, 12, 24, and 48 h	Novafen was superior to Gelofen and placebo at 8 h. There was no significant difference among groups at 4, 12, 24, and 48 h. Pain at 4 h: *p*‐value = 0.16 Pain at 8 h: *p*‐value = 0.03 Pain at 12 h: *p*‐value = 0.18 Pain at 24 h: *p*‐value = 0.95 Pain at 48 h *p*‐value = 0.59
Flath et al. [65] (4)	P: 20–80 years, mean age 49 years I1: flurbiprofen 100 mg oral pretreatment + placebo postoperative (*n* = 30) I2: flurbiprofen 200 mg oral (100 mg pretreatment + 100 mg oral postoperative) (*n* = 30) I3: placebo (pretreatment) + flurbiprofen 100 mg oral postoperative (*n* = 30) C: placebo (pretreatment and postoperative) (*n* = 30) O: reduction in pain (VAS) Follow‐up: 3, 7, and 24 h	Flurbiprofen 100 mg (pretreatment) was superior to placebo at 7 and 24 h (*p*‐value <0.05).
Isik et al. [66] (2)	P: 18–45 years I1: gabapentin 600 mg oral (*n* = 30) I2: lornoxicam 8 mg oral (*n* = 30) C: placebo (*n* = 30) O: reduction in pain (VAS) Follow‐up: 4, 8, 12, and 24 h	There was no significant difference among groups at 4 or 8 h. Gabapentin was superior to placebo (*p*‐value < 0.0001) or lornoxican (*p*‐value < 0.008) at 12 h. Gabapentin was superior to placebo (*p*‐value = 0.001) at 24 h. Lornoxican was superior to placebo (*p*‐value <0.0001) at 12 h. Pain at 4 h: *p*‐value = 0.091 Pain at 8 h: *p*‐value = 0.334 Pain at 12 h: *p*‐value <0.0001 Pain at 24 h: *p*‐value = 0.003
Khorasani and Mahmudi [49] (2)	P: 25–50 years I1: ibuprofen 400 mg oral (*n* = 16) I2: sulindac 200 mg oral (*n* = 16) C: placebo (*n* = 16) O: reduction in pain (VAS) Follow‐up: 6, 12, 24, 48, and 72 h	There was no significant difference among groups at 6, 12, 24, 48, and 72 h (*p*‐value > 0.05).
Gopikrishna and Parameswaran [14] (6)	P: 18–65 years I1: rofecoxib 50 mg oral (*n* = 15) I2: ibuprofen 600 mg oral (*n* = 15) C: placebo (*n* = 15) O: reduction in pain (VAS) Follow‐up: 4, 8, 12, 24, 48, and 72 h	Rofecoxib or ibuprofen was superior to placebo at 4 and 8 h (*p*‐value <0.05). There was no significant difference between rofecoxib and ibuprofen during both 4 h (*p*‐value = 0.30) and 8 h (*p*‐value = 0.25). Rofecoxib was superior to ibuprofen and placebo at 12 h (*p*‐value = 0.003) and 24 h (*p*‐value = 0.047).
Mokhtari et al. [52] (6)	P: 19–30 years I1: ibuprofen 400 mg oral (*n* = 22) I2: indomethacin 25 mg oral (*n* = 22) C1: placebo (*n* = 22) O: reduction in pain (VAS) Follow‐up: 8, 12, and 24 h	Indomethacin or ibuprofen was superior to placebo at 8 h (*p*‐value = 0.001 and *p*‐value <0.001, respectively). There was no significant difference between the three groups at 12 h (*p*‐value = 0.80) and 24 h (*p*‐value = 0.27).

Abbreviations: IL, intraligamentary; IM, intramuscular; NNT, number needed to treat; NRS, numeric rating scale; RCT, randomized controlled trial; RRR, relative risk reduction; SR, systematic review; VAS, visual analog scale.

Three meta‐analyses evaluated the pretreatment NSAIDs in reducing postoperative pain in adult patients after nonsurgical root canal treatment [31, 34, 35].

De Geus et al. [31] compared ibuprofen at various concentrations (200, 400, or 600 mg) versus other drugs (tenoxicam 20 mg, rofecoxib 50 mg, indomethacin 25 mg, and zintoma 2 g) or placebo, at 6–8 or 24 h. Ibuprofen was not superior to placebo or other drugs. In a sensitivity analysis including five RCTs, ibuprofen was superior to placebo at 24 h (SMD = −0.61; 95% CI: −1.05 to −0.17). The quality of the evidence was low or very low for all measures.

Kumar et al. [34] performed a meta‐analysis with two RCTs comparing the oral NSAIDs versus control at 6, 12, or 24 h and parenteral NSAIDs versus control at 24 h. Oral NSAIDs were superior to control at 6 h (MD = −0.48; 95% CI: −0.91 to −0.05) and parenteral NSAIDs were superior at 24 h (MD = −0.65; 95% CI: −1.01 to −0.29). Oral NSAIDs showed no significant difference with the control after 12 or 24 h. The quality of evidence was low or very low for all measures, assessed by pooling of RCTs with NSAIDs and corticosteroids in both routes.

One study [35] compared ibuprofen with placebo at 6, 12, and 24 h. Meta‐analysis with three RCTs also found that ibuprofen was not superior to placebo. The authors conducted a meta‐analysis comparing NSAIDs versus placebo at 6 h (4 RCTs), 12, or 24 h (6 RCTs). NSAIDs were not significantly superior to placebo. The quality of evidence was very low for NSAIDs measures (at 6, 12, and 24 h) and was not reported for ibuprofen.

Four systematic reviews with meta‐analyses assessed the use of corticosteroids in reducing postoperative pain in adult patients after nonsurgical root canal treatment [34–36, 40].

Kumar et al. [34] performed a meta‐analysis with two or more RCTs comparing pretreatment oral and parenteral corticosteroids versus control at 6, 12, and 24 h. Three RCTs were included in the meta‐analysis and confirmed that oral corticosteroids were superior to control at 6 h (MD = −0.99; 95% CI: −1.71 to −0.28), 12 h (MD = −1.19; 95% CI: −1.95 to −0.44), and 24 h (MD = −1.39; 95% CI: −2.69 to −0.10). Meta‐analysis with two RCTs also confirmed the superiority of parenteral corticosteroids over control at 6 h (MD = −1.31; 95% CI: −2.18 to −0.44), 12 h (MD = −0.94; 95% CI: −1.33 to −0.56), and 24 h (MD = −0.71; 95% CI: −1.02 to −0.43). The quality of evidence was low or very low for all measures; however, it was assessed by pooling RCTs with NSAIDs and corticosteroids, oral and parenteral.

Nagendrababu et al. [35] compared corticosteroids with placebo at 6, 12, and 24 h. Three RCTs with prednisolone were included, and the meta‐analysis showed that prednisolone was superior to placebo at 6 h (MD = −20.18; 95% CI: −36.54 to −3.82), 12 h (MD = −20.43; 95% CI: −28.22 to −12.64), and 24 h (MD = −21.23; 95% CI: −36.42 to −6.04). Suneelkumar et al. [40] had already conducted a meta‐analysis with the same three RCTs included in Nagendrababu et al. [35]. The quality of evidence was very low for measures at 6 and 24 h and moderate at 12 h.

Nath et al. [36] conducted a meta‐analysis comparing postoperative pain reduction with corticosteroids versus placebo at 4–6, 12, or 24 h. Corticosteroids were superior to placebo at 4–6 h (MD = −21.15; 95% CI: −35.03 to −7.26) or 12 h (MD = −19.57; 95% CI: −34.77 to −4.37), while at 24 h, there was no significant difference. Subgroup analyses found that intraligamentary (IL) administration of corticosteroids was superior to placebo between 4–24 h (MD = −25.17; 95% CI: −34.87 to −15.46) and oral corticosteroids were superior to placebo in reducing postoperative pain at 12 h (MD = −19.63; 95% CI: −37.07 to −2.19). Dexamethasone (oral 4 mg or IL injection 0.2 mL) was not significantly superior to placebo at 4–6 h, but oral prednisolone was superior to placebo at 4–6 h (MD = −23.07; 95% CI: −40.37 to −5.76). The quality of evidence was low for corticosteroid measures at 4–6 and 24 h. Two other outcomes evaluated were not restricted to pretreatment use.

### 3.3. RCT Included in Selected Systematic Reviews

A total of 114 primary study citations about pretreatment corticosteroids or NSAIDs were identified across 14 systematic reviews, representing 36 unique RCTs. The calculated CCA was 15.9%, indicating a very high degree of overlap among the included reviews. For systematic reviews of corticosteroids, the calculated CCA was 30.0%, whereas for NSAIDs, it was 14.6%. The overlap of RCTs in systematic reviews included and CCA can be found in Supporting Information 3: Table S3.

Eleven RCTs included in selected systematic reviews evaluated pretreatment corticosteroids (Table 3) and 28 evaluated pretreatment NSAIDs (Table 4). Dexamethasone (*n* = 7), prednisolone (*n* = 3), deflazacort (*n* = 1), and methylprednisolone (*n* = 1) were compared with placebo, NSAIDs, opioids, and among themselves. Ibuprofen (*n* = 14), ketorolac (*n* = 6), diclofenac (*n* = 5), piroxicam (*n* = 3), celecoxib (*n* = 2), and others NSAIDs (*n* = 7) were compared with placebo, corticosteroids, and among.

#### 3.3.1. Pretreatment Corticosteroids

Dexamethasone 4 mg (*n* = 4) and 8 mg (*n* = 1) were evaluated by oral, IL (*n* = 2), supraperiosteal (*n* = 1), or intramuscular (IM) (*n* = 1) routes compared with placebo, ibuprofen, or piroxicam. Dexamethasone showed a significant reduction in pain after 4–6 h (in 6 of 7 RCTs that evaluated this time), 12 h (in 5 of 6 RCTs), and 24 h (in 4 of 6 RCTs) when compared with placebo. Oral dexamethasone 8 mg compared with placebo and ibuprofen found no difference between groups in postoperative pain reduction after 4, 8, 12, 24, or 48 h [10]. In two other studies with oral dexamethasone (4 mg), there was no significant difference in postoperative pain reduction after 24 h [19, 42].

Oral prednisolone 30 or 40 mg significantly reduced post‐endodontic pain at 12 and 24 h when compared with placebo [7, 11, 44]. One study [11] found that this effect lasted up to 48 h. Prednisolone was significant in reducing the postoperative pain score at 6 h in two studies [7, 44].

One RCT [45] evaluated the administration of slow‐release methylprednisolone 4–8 mg IL versus placebo. The methylprednisolone IL was superior to placebo at 24 h.

Another study [20] compared deflazacort 30 mgwith piroxicam 20 mg, dexamethasone 4 mg, and placebo by an oral route. The three anti‐inflammatories were superior to placebo at 6, 12, and 24 h and showed no statistically significant difference between them. There was also no difference between the groups at 48 and 72 h.

#### 3.3.2. Pretreatment NSAIDs

Fourteen studies evaluated the use of ibuprofen in doses of 200, 400, and 600 mg in tablets or capsules. Ibuprofen was superior to placebo at 2–8 h in seven studies [14, 42, 46, 48, 50, 52, 54], while in three studies [11, 47, 51], there was no statistically significant difference at 4 h. Four RCTs [10, 16, 49, 53] did not find a statistically significant difference between ibuprofen and placebo at any of the times evaluated (6–72 h). A study [54] that compared ibuprofen, slow‐released diclofenac sodium, and placebo proved that ibuprofen was superior to placebo at 2, 10, 18, and 36 h and to diclofenac sodium at 2 h. Slow‐released diclofenac sodium was superior to ibuprofen at 10, 18, and 36 h and to placebo at 2, 10, 18, and 36 h. The fixed‐dose combination ibuprofen 200 mg + acetaminophen 325 mg + caffeine 40 mg (oral) (Novafen) was superior to oral ibuprofen 400 mg (Gelofen) and placebo at 8 h [47]. Bali et al. [15] compared ibuprofen 200 mg, tenoxicam 20 mg, and ketorolac 10 mg at 6–72 h and found no statistically significant difference between the groups at 6 h. However, after 6 h follow up, ibuprofen 200 mg and ketorolac 10 mg did not exhibit a statistically significant difference, and they were superior to tenoxicam 20 mg.

Two RCTs [55, 56] evaluated the use of ketorolac IL 30 mg in 1 mL; three RCTs by oral route 10 mg [15, 58] or 20 mg [11]; and Penniston and Hargreaves [57] compared ketorolac 30 mg by the IM route and periapical infiltration. Ketorolac IL presented divergent results in the two studies identified, and it was not recommended because the technique used caused pain in four out of five patients [56]. In a study [58] comparing oral ketorolac 10 mg, tapentadol, and etodolac, both ketorolac and tapentadol demonstrated superiority over etodolac at all measured time points (6, 12, 18, and 24 h). Furthermore, ketorolac exhibited superiority over tapentadol specifically at 6, 12, and 18 h. Oral ketorolac 20 mg was superior to placebo and oral prednisolone 30 mg at 6 h. However, prednisolone was superior to placebo and ketorolac at 12, 24, and 48 h [11].

Two RCTs [59, 61] evaluated diclofenac sodium IL and found that it was superior to placebo in reducing postoperative pain within 4–48 h. The IL route was superior to the oral route up to 48 h. Oral diclofenac sodium 75 mg was superior to placebo at 6, 12, 24, and 48 h [59]. Another RCT [60] that compared oral diclofenac sodium 100 mg and placebo demonstrated that the intervention was superior to placebo at 6 and 12 h, with no statistically significant difference at 24 h. Slow‐released diclofenac sodium 100 mg was superior to ibuprofen at 10, 18, and 36 h [54]. Oral diclofenac potassium 50 mg was superior to placebo only at 48, with no statistically significant difference at 6, 12, and 24 h [9].

Two studies compared oral etodolac 400 mg with ibuprofen, tapentadol, ketorolac, and placebo. Oral ibuprofen 600 mg was superior to etodolac at 4 and 8 h [50]; ketorolac and tapentadol demonstrated superiority over etodolac at 6, 12, 18, and 24 h [58].

Three RCTs evaluated pretreatment with piroxicam. Two studies that evaluated piroxicam IL 20 mg/mL found that it was superior to the control group for periods of up to 48 h [62, 63]. A study that compared oral piroxicam 20 mg, dexamethasone 4 mg, and deflazacort 30 mg to placebo found that the three medications were superior to placebo at 6, 12, and 24 h. No significant difference was observed between the groups in any follow‐ups and between them and placebo at 48 and 72 h [20]. Joshi et al. [63] compared oral piroxicam 40 mg to piroxicam IL 20 mg/mL. There was no significant difference between oral piroxicam and IL at 4 and 8 h. Piroxicam IL was superior to the oral route at 12, 24, and 48 h.

Ashraf et al. [64] compared oral celecoxib 400 mg to placebo and found no statistically significant difference between the groups at 4, 8, 12, 24, and 48 h. Another RCT compared oral celecoxib 200 mg to oral ibuprofen 400 mg and placebo and found that celecoxib and ibuprofen were superior to placebo at 8 and 12 h, with no significant difference between the groups at 4 and 24 h [51].

Seven RCTs evaluated other NSAIDs (tenoxicam, Novafen [ibuprofen 200 mg + acetaminophen 325 mg + caffeine 40 mg oral route], flurbiprofen, lornoxicam, sulindac, rofecoxib, and indomethacin). Oral tenoxicam 20 mg showed inferior results to oral ibuprofen 200 mg and oral ketorolac 10 mg at 12, 24, 48, and 72 h. Since ketorolac and tenoxicam have better gastrointestinal tolerance, they should be preferred over the other two in controlling post‐endodontic pain [15]. Novafen (ibuprofen 200 mg + acetaminophen 325 mg + caffeine 40 mg) was superior to Gelofen (ibuprofen 400 mg) and placebo at 8 h, and there was no significant difference among groups at 4, 12, 24, and 48 h [47]. Pretreatment oral flurbiprofen 100 mg [65] was superior to placebo at 7 and 24 h. Oral lornoxicam 8 mg was compared to oral gabapentin 600 mg and placebo [66]. There was no significant difference between groups at 4 or 8 h. Lornoxicam was superior to placebo at 12 h. Oral sulindac 200 mg was compared to oral ibuprofen 400 mg and placebo. There was no significant difference among groups at 6, 12, 24, 48, and 72 h [49]. Oral rofecoxib 50 mg compared to oral ibuprofen 600 mg and placebo showed superior results to placebo at 4 and 8 h and no difference with ibuprofen at the same times; however, rofecoxib was superior to ibuprofen and placebo at 12 and 24 h [14]. Oral indomethacin 25 mg was superior to placebo at 8 h. There was no significant difference between indomethacin oral ibuprofen 400 mg and placebo at 12 and 24 h [52].

### 3.4. Methodological Quality Assessment

Regarding the methodological quality assessment, most of the systematic reviews included had at least two critical flaws. These flaws were mainly related to the absence of a description of a protocol prior to the study, the lack of explanation for the selection criteria for the designs of the included studies, not providing the list of excluded studies and the reasons for exclusions, not informing about funding sources for clinical trials, and not taking into account the risk of bias when discussing the results of the review (Table 5).

**Table 5:** Methodological quality assessment of systematic reviews included (*n* = 14).

Systematic reviews	1	2^a^	3	4^a^	5	6	7^a^	8	9^a^	10	11^a^	12	13^a^	14	15^a^	16
Almuthhin et al. [13]	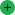	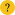	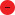	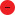	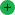	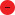	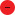	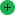	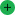	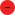	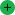	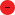	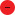	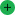	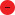	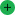
Aminoshariae et al. [30]	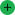	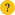	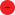	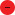	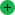	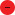	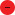	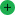	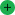	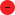	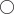	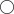	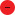	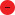	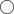	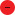
De Geus et al. [31]	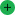	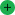	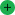	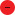	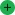	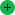	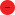	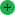	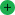	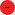	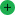	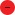	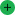	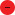	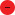	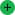
Iranmanesh et al. [32]	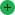	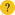	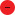	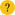	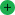	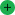	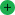	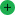	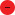	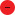	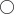	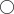	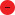	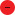	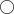	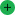
Jose et al. [33]	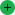	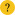	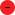	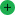	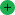	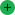	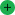	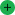	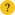	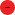	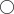	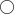	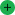	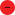	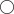	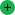
Kumar et al. [34]	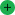	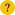	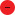	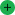	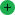	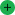	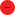	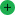	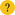	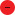	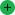	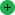	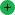	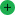	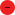	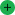
Nagendrababu et al. [35]	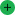	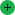	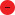	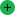	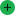	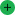	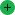	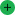	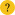	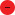	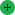	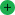	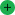	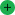	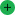	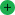
Nath et al. [36]	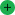	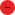	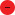	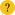	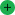	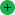	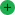	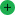	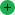	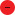	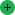	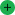	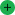	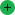	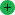	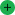
Santini et al. [37]	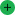	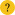	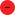	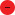	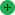	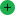	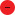	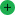	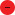	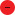	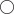	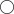	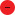	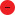	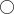	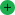
Shamszadeh et al. [23]	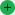	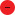	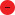	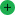	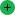	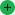	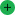	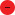	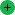	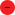	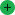	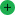	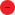	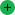	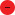	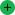
Shirvani et al. [38]	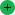	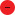	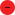	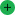	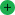	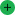	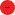	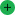	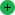	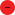	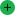	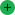	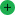	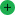	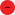	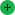
Smith et al. [39]	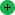	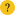	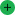	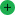	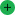	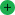	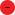	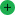	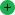	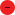	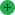	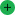	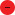	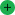	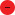	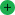
Suneelkumar et al. [40]	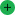	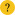	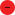	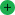	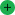	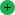	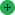	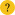	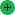	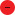	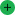	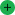	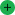	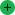	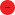	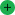
Teja et al. [22]	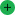	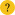	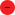	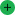	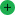	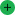	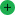	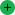	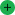	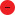	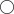	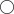	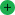	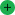	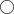	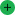

*Note:* 1. Did the research questions and inclusion criteria for the review include the components of PICO? 2. Did the report of the review contain an explicit statement that the review methods were established prior to the conduct of the review and did the report justify any significant deviations from the protocol? 3. Did the review authors explain their selection of the study designs for inclusion in the review? 4. Did the review authors use a comprehensive literature search strategy? 5. Did the review authors perform study selection in duplicate? 6. Did the review authors perform data extraction in duplicate? 7. Did the review authors provide a list of excluded studies and justify the exclusions? 8. Did the review authors describe the included studies in adequate detail? 9. Did the review authors use a satisfactory technique for assessing the risk of bias (RoB) in individual studies that were included in the review? 10. Did the review authors report on the sources of funding for the studies included in the review? 11. If meta‐analysis was performed, did the review authors use appropriate methods for statistical combination of results? 12. If meta‐analysis was performed, did the review authors assess the impact of RoB in individual studies on the results of the meta‐analysis or other evidence synthesis? 13. Did the review authors account for RoB in primary studies when interpreting/discussing the results of the review? 14. Did the review authors provide a satisfactory explanation for, and discussion of, any heterogeneity observed in the results of the review? 15. If they performed quantitative synthesis did the review authors carry out an adequate investigation of publication bias and discuss its likely impact on the results of the review? 16. Did the review authors report any potential sources of conflict of interest, including any funding they received for conducting the review? 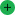 Yes 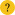 Partial Yes 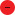 No 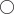 No meta‐analyses conducted.

^a^Issues considered by AMSTAR to be critically important.

### 3.5. Report on Other Outcomes: Adverse Drug Reactions, Rescue Medication Use, and Quality of Life

Nine systematic reviews presented some information on adverse events, and six systematic reviews addressed the use of rescue medication related to the pretreatment use of analgesics and anti‐inflammatories in pain management for patients undergoing nonsurgical endodontic treatment. The information obtained from the systematic reviews was examined in the respective primary studies and is reported as originally found.

#### 3.5.1. Adverse Drug Reactions

##### 3.5.1.1. Corticosteroids

Prednisolone has no reported side effects [7, 44]. This finding was supported by three systematic reviews [35, 37, 40].

##### 3.5.1.2. NSAIDs

The adverse effects reported for etodolac were nausea, vomiting, headache, dizziness, and heartburn, whereas ketorolac studies reported headache and dizziness [58]. In Bali et al. [15], ketorolac did not present any adverse events. One study [66] with lornoxicam reported gastrointestinal pain. Indomethacin [52], ibuprofen [15, 17, 46, 47, 49, 52], and tenoxicam [15, 46] had no reports of any side effects. The fixed‐dose combination of ibuprofen 200 mg + acetaminophen 325 mg + caffeine 40 mg PO (Novafen) also did not present any adverse reactions [47]. In the study by Flath et al. [65], 7 of the 28 volunteers who used pretreatment flurbiprofen presented dizziness, drowsiness, and lightheadedness; gastrointestinal symptoms, including upset stomach, constipation, and flatulence; or other adverse effects (xerostomia, feeling warm, tachycardia, itching, sweating, rash, wheezing, or tightness in the chest). In Praveen et al. [11], volunteers were informed about the possibility of drug interactions between corticosteroids and NSAIDs. However, no occurrences were observed in this study.

#### 3.5.2. Rescue Medication Use

##### 3.5.2.1. Corticosteroids

In the study by Jalalzadeh et al. [44], 6 out of 20 patients in the prednisolone group and 8 out of 20 in the control group used rescue medication and were subsequently excluded from the analysis. In Kaufmann et al. [45], three patients who reported severe postoperative pain used rescue analgesics and were excluded from the analysis. The authors did not report which group these patients belonged to: methylprednisolone, mepivacaine (active placebo), or no drug (passive placebo). Sharma et al. [43] studied the pretreatment dexamethasone given through oral, IM, supraperiosteal, and IL routes and established the exclusion of patients using rescue medication (acetaminophen); however, they did not report whether this occurred. In the Elkhaden et al. [7] trial, a sham capsule was used initially as a rescue medication not to mask the initial postoperative pain intensity, followed by analgesic intake if the patient’s symptoms persisted. As for the sham or analgesic intake following single‐visit root canal treatment, prednisolone provided significantly less need for rescue medication, whether sham or analgesic. The reduction in the relative risk of the need for rescue medication with prednisolone was more than half (54% for the need for sham and 55% for the need for analgesics, respectively) compared to that required for the control group. Patients who took it would be reported but not eliminated from the trial as an “intention‐to‐treat analysis” was used in this study. In Praveen et al. [11], six patients were excluded from the study due to using rescue medication (ibuprofen): 2/31 in the ketorolac group, 1/31 in the prednisolone group, and 3/30 in the placebo group.

##### 3.5.2.2. NSAIDs

In the study by Ashraf et al. [64], 10 out of 15 patients in the placebo group and 6 out of 15 in the celecoxib group used rescue medication. In Menke et al. [50], 22 patients (61%) needed additional analgesics (the same for each group—ibuprofen or etodolac). It was not specified to which group these patients belonged. In Bali et al. [15] [ibuprofen, tenoxicam, and ketorolac], Arslam et al. [46] [tenoxicam and ibuprofen], and Attar et al. [16] [ibuprofen tablet or liquigel], no patient used the intended rescue medication. Gopikrishna and Parameswaran [14] studied the pretreatment with rofecoxib and ibuprofen to establish if any relationship exists between periapical diagnosis and the need for additional medication after completion of pulpectomy, observing that patients with a diagnosis of acute apical periodontitis were more likely to require additional medication for postendodontic pain than those with a periapical diagnosis of a normal periapex, chronic apical periodontitis, or chronic apical abscess. In Mello [42], the placebo group showed a significantly higher consumption of analgesics (69.7%) than the dexamethasone (6.1%) and ibuprofen (19.3%) groups. Mehrvarzfar et al. [41] also found a statistically significant difference in the consumption of rescue analgesics: the dexamethasone and lidocaine groups showed lower consumption than the placebo group. Mokthari et al. [52] recommended the use of additional analgesics, if necessary, but did not report whether anyone used them. In Ramazani et al. [53], the patients who had to take analgesics other than acetaminophen because of intense pain or any other reason were omitted from the study and replaced with other ones. There was no significant statistical difference observed because of taking acetaminophen by the patients in the three groups [ibuprofen, Zintoma (a derivative of ginger), and placebo (*p* > 0.05)]. One patient in the tapentadol group (*n* = 20) and one in the ketorolac group (*n* = 20) used rescue medication and were excluded from the study [58].

#### 3.5.3. Quality of Life

No systematic review addressed the quality of life.

## 4. Discussion

### 4.1. Main Findings

This overview of systematic reviews synthesized the available evidence on pretreatment anti‐inflammatory and analgesic drugs for pain management in patients undergoing nonsurgical endodontic treatment. The included reviews focused mainly on NSAIDs and corticosteroids, whereas no review exclusively evaluated pretreatment opioid or nonopioid analgesics. Oral and parenteral routes were the most frequently investigated, and six systematic reviews conducted meta‐analyses, including five pairwise meta‐analyses and one network meta‐analysis.

Overall, the quantitative evidence from systematic reviews favored pretreatment corticosteroids over placebo for reducing postoperative pain at 6, 12, and 24 h, particularly with prednisolone, although some divergence remained at 24 h, and the certainty of evidence was predominantly low or very low. In contrast, findings for NSAIDs were more heterogeneous and often inconsistent across reviews, with only limited convergent evidence supporting piroxicam. The methodological quality of the included systematic reviews was predominantly low, with most being rated as critically low by AMSTAR 2, which substantially limits confidence in these findings.

Given the substantial clinical and methodological heterogeneity across the primary trials, many studies could not be included in meta‐analyses, and a more detailed examination of the RCT evidence was necessary. Among corticosteroids, dexamethasone was the most frequently studied agent in the primary studies, administered orally or by IL, supraperiosteal, or IM routes, but its findings were not fully consistent across studies despite a general trend toward pain reduction within the first 24 h. By contrast, oral prednisolone (30–40 mg) showed the most consistent favorable results, with superiority over placebo at 12 and 24 h and, in most studies, also at 6 h. Deflazacort and slow‐release IL methylprednisolone were each evaluated in only one RCT, but both showed favorable results compared with placebo.

Among NSAIDs, piroxicam was the only drug to show relatively convergent findings across primary studies, with favorable effects versus placebo at 6, 12, and 24 h and better performance of the IL route over the oral route at later time points. In contrast, ibuprofen, although the most frequently studied NSAID, yielded conflicting findings in both systematic reviews and primary trials. A similar inconsistency was observed for ketorolac, diclofenac, and celecoxib. Other NSAIDs, including tenoxicam, flurbiprofen, indomethacin, lornoxicam, sulindac, rofecoxib, and the fixed‐dose combination of ibuprofen, acetaminophen, and caffeine, were each assessed in isolated RCTs and showed variable results, precluding any robust inference regarding superiority.

Taken together, these findings suggest that pretreatment corticosteroids, particularly prednisolone, may be associated with more consistent short‐term analgesic benefits than NSAIDs after nonsurgical endodontic treatment. These findings, however, should be interpreted considering the predominantly critically low methodological quality of the included reviews and the substantial heterogeneity across the underlying trials, both of which limited the reliability and clinical applicability of the available evidence.

Adverse drug reactions and rescue medication use were addressed in only part of the included systematic reviews and primary studies, with generally limited and inconsistent reporting. By contrast, quality of life was not assessed in any of the 14 systematic reviews included or the 36 unique primary studies. This absence should be regarded as an important gap in the current literature, reflecting the prevailing emphasis on pain intensity rather than on broader patient‐centered outcomes.

### 4.2. Strengths and Limitations of the Study

This overview of systematic reviews included a comprehensive search across seven databases. Study selection, data extraction, and methodological quality assessment were conducted independently by peer reviewers. The findings are based on the information reported in the included reviews and may therefore reflect the methodological limitations of the original studies.

The present synthesis provides an integrative and critical synthesis of a fragmented body of evidence by bringing together data from 14 systematic reviews and 36 RCTs included across those reviews. Although a high degree of overlap among reviews was observed, no de novo meta‐analysis was conducted; consequently, the overlap did not result in statistical double‐counting.

Beyond summarizing the available evidence, this work also identifies consistencies and discrepancies across reviews, making explicit the substantial clinical and methodological heterogeneity in terms of drug classes, dosages, routes and timing of administration, comparators, pain assessment scales, and follow‐up time points. By examining both the limitations of the primary studies and the methodological shortcomings of the included systematic reviews, this overview moves beyond simple aggregation and provides a higher‐level appraisal of the field, allowing for the identification of unresolved questions and the formulation of recommendations for future research.

Some systematic reviews evaluated drug administration at different time points (before, during, and after treatment). Regardless of the scope of these reviews, data extraction in this overview was restricted exclusively to pretreatment administration in accordance with predefined eligibility criteria. In addition, some primary studies were misclassified as pretreatment in the original reviews and were excluded after verification.

A key limitation of the available evidence is the insufficient reporting of the baseline clinical status of treated teeth. Diagnostic categories such as irreversible pulpitis or apical periodontitis were inconsistently described, precluding reliable stratification of the results. This limits clinical interpretability and may contribute to heterogeneity across studies. Retreatment cases are outside the scope of this overview. Future research should ensure standardized diagnostic reporting to enhance applicability.

No language restrictions were applied to the inclusion of systematic reviews. However, during the analysis of underlying primary studies, some articles could not be retrieved or were excluded due to language barriers (e.g., Persian). This limitation is related to the extraction of data from RCTs rather than to the eligibility criteria of systematic reviews, but it may have introduced a certain degree of selection bias.

### 4.3. Implications for Clinical Practice and Research

The pretreatment use of anti‐inflammatory and analgesic drugs for pain management in patients undergoing non‐surgical endodontic treatment has been widely investigated and frequently applied in clinical endodontic practice as a strategy to reduce postoperative pain, particularly in patients with symptomatic inflammatory conditions, but there is still no universally established gold‐standard protocol. Drugs from different classes have been used to seek better clinical outcomes, with corticosteroids and NSAIDs being the most frequently investigated.

Studies evaluating pretreatment corticosteroids have generally suggested favorable effects on pain reduction in patients undergoing endodontic treatment, particularly with prednisolone. NSAIDs, in contrast, have yielded inconsistent findings, and only one systematic review with network meta‐analysis [35] found evidence in favor of the use of piroxicam. These findings were also supported by the analysis of the primary studies [20, 62, 63].

Although the available evidence is heterogeneous, the relatively more consistent short‐term findings for corticosteroids may be biologically plausible. In endodontic inflammatory conditions, corticosteroids inhibit phospholipase A2 and modulate inflammatory cytokine pathways upstream in the arachidonic acid cascade, while NSAIDs primarily act through cyclooxygenase inhibition and the consequent reduction of prostaglandin synthesis. This broader anti‐inflammatory effect, particularly when administered before instrumentation, may contribute to earlier attenuation of inflammatory mediator release and partially explain the clinical findings observed in some studies.

The marked heterogeneity among the included clinical trials (evaluating different populations, clinical conditions, procedures, drugs, doses, routes of administration, follow‐up times, and outcomes) limited the feasibility of meta‐analyses in several reviews and hindered the definition of consistent clinical protocols for pretreatment analgesia in endodontics. Therefore, these findings should be interpreted cautiously, with emphasis on individualized clinical judgment and the need for higher‐quality evidence to support stronger recommendations.

This overview also underscores the need for rigorously designed clinical trials capable of providing robust evidence to determine the most effective and safe anti‐inflammatory and analgesic agents, along with their optimal doses and routes of administration, for pain management in patients undergoing nonsurgical endodontic treatment.

## 5. Conclusions

The pretreatment use of anti‐inflammatory and analgesic drugs to reduce pain in patients undergoing nonsurgical endodontic treatment has been studied for a long time but without assertive conclusions. Few systematic reviews were able to perform meta‐analyses due to the heterogeneity (population, procedures, medications, doses, routes of administration, follow‐up times, and outcomes) of the primary studies. The methodological quality of systematic reviews and primary studies also limits the definition of clinical protocols.

Pretreatment corticosteroids appeared to be associated with more consistent reductions in postoperative pain among patients undergoing endodontic treatment; however, this observation is limited by the critically low quality of most included reviews and by heterogeneity and imprecision in the available evidence. Evidence on NSAIDs remains inconsistent, and studies on opioid analgesics are scarce. Further high‐quality clinical trials are needed before firm clinical recommendations can be established.

This investigation can guide future scientific studies that facilitate the development of clinical protocols for the effective and safe pretreatment use of analgesics and anti‐inflammatories in endodontics.

## Author Contributions

**Cristiane Midori Takahashi:** conceptualization, investigation writing – original draft preparation. **Régis Penha Pimenta:** investigation, validation. **Cristiane de Cássia Bergamaschi:** supervision, methodology. **Fabio da Silva Moraes**: investigation, validation. **Silvio Barberato-Filho:** supervision, methodology, writing – review & editing.

## Funding

This study did not receive any specific funding. Cristiane Midori Takahashi and Régis Penha Pimenta received doctoral scholarships from the University of Sorocaba.

## Disclosure

All authors have read and approved the final version of the manuscript. Silvio Barberato‐Filho had full access to all the data in this study and takes complete responsibility for the integrity of the data and the accuracy of the data analysis.

## Ethics Statement

The protocol of this systematic review was registered in the International Prospective Register of Systematic Reviews (PROSPERO), under #ID CRD42022355785.

## Conflicts of Interest

The authors declare no conflicts of interest.

## Supporting Information

Additional supporting information can be found online in the Supporting Information section.

## Supporting information


**Supporting Information 1** Table S1: Search strategies.

**Supporting Information 2** Table S2: Excluded studies.

**Supporting Information 3** Table S3: Overlap of primary studies (RCT) in systematic reviews included and corrected covered area (CCA).

## Data Availability

The authors confirm that the data supporting the findings of this study are available within the article and/or its supporting information.

## References

[1] Kumar U. , Parmar P. , Vashisht R. , Tandon N. , and Kaur C. K. , Incidence of Postoperative Pain After Using Single Continuous, Single Reciprocating, and Full Sequence Continuous Rotary File System: A Prospective Randomized Clinical Trial, Journal of Dental Anesthesia and Pain Medicine. (2023) 23, no. 2, 91–99, 10.17245/jdapm.2023.23.2.91. 37034837 PMC10079766

[2] Montero J. , Lorenzo B. , Barrios R. , Albaladejo A. , Mirón Canelo J. A. , and López-Valverde A. , Patient-Centered Outcomes of Root Canal Treatment: A Cohort Follow-up Study, Journal of Endodontics. (2015) 41, no. 9, 1456–1461, 10.1016/j.joen.2015.06.003. 26211565

[3] Gotler M. , Bar-Gil B. , and Ashkenazi M. , Postoperative Pain After Root Canal Treatment: A Prospective Cohort Study, International Journal of Dentistry. (2012) 2012, 5, 10.1155/2012/310467, 310467. 22505897 PMC3312224

[4] Pak J. G. and White S. N. , Pain Prevalence and Severity Before, During, and After Root Canal Treatment: A Systematic Review, Journal of Endodontics. (2011) 37, no. 4, 429–438, 10.1016/j.joen.2010.12.016. 21419285

[5] Arias A. , de la Macorra J. C. , Hidalgo J. J. , and Azabal M. , Predictive Models of Pain Following Root Canal Treatment: A Prospective Clinical Study, International Endodontic Journal. (2013) 46, no. 8, 784–793, 10.1111/iej.12059. 23402273

[6] Nagendrababu V. and Gutmann J. L. , Factors Associated With Postobturation Pain Following Single-Visit Nonsurgical Root Canal Treatment: A Systematic Review, Quintessence International. (2017) 48, no. 3, 193–208, 10.3290/j.qi.a36894. 27669726

[7] Elkhadem A. , Ezzat K. , and Ramadan M. , et al.The Effect of Preoperative Oral Administration of Prednisolone on Postoperative Pain in Patients With Symptomatic Irreversible Pulpitis: A Single-Centre Randomized Controlled Trial, International Endodontic Journal. (2018) 51, no. S3, 10.1111/iej.12795. 28560802

[8] Aksoy F. and Ege B. , The Effect of Pretreatment Submucosal Injections of Tramadol and Dexamethasone on Post-Endodontic Pain in Mandibular Molar Teeth With Symptomatic Irreversible Pulpitis: A Randomized Controlled Clinical Trial, International Endodontic Journal. (2020) 53, no. 2, 176–185, 10.1111/iej.13246. 31702056

[9] Al-Rawhani A. H. , Gawdat S. I. , and Wanees Amin S. A. , Effect of Diclofenac Potassium Premedication on Postendodontic Pain in Mandibular Molars With Symptomatic Irreversible Pulpitis: A Randomized Placebo-Controlled Double-Blind Trial, Journal of Endodontics. (2020) 46, no. 8, 1023–1031, 10.1016/j.joen.2020.05.008. 32470370

[10] Jorge-Araújo A. C. A. , Bortoluzzi M. C. , Baratto-Filho F. , Santos F. A. , and Pochapski M. T. , Effect of Premedication With Anti-Inflammatory Drugs on Post-Endodontic Pain: A Randomized Clinical Trial, Brazilian Dental Journal. (2018) 29, no. 3, 254–260, 10.1590/0103-6440201801786. 29972451

[11] Praveen R. , Thakur S. , and Kirthiga M. , Comparative Evaluation of Premedication With Ketorolac and Prednisolone on Postendodontic Pain: A Double-Blind Randomized Controlled Trial, Journal of Endodontics. (2017) 43, no. 5, 667–673, 10.1016/j.joen.2016.12.012. 28320541

[12] Suresh N. , Nagendrababu V. , and Koteeswaran V. , et al.Effect of Preoperative Oral Administration of Steroids in Comparison to an Anti-Inflammatory Drug on Postoperative Pain Following Single-Visit Root Canal Treatment—A Double-Blind, Randomized Clinical Trial, International Endodontic Journal. (2021) 54, no. 2, 198–209, 10.1111/iej.13416. 32976660

[13] Almuthhin M. , Afify M. , and Alshammari Y. , et al.The Safety and Efficacy of Pre- and Post-Medication for Postoperative Endodontic Pain: A Systematic Review and Network Meta-Analysis, The Open Dentistry Journal. (2020) 14, no. 1, 563–599, 10.2174/1874210602014010563.

[14] Gopikrishna V. and Parameswaran A. , Effectiveness of Prophylactic use of Rofecoxib in Comparison With Ibuprofen on Postendodontic Pain, Journal of Endodontics. (2003) 29, no. 1, 62–64, 10.1097/00004770-200301000-00017. 12540224

[15] Bali R. , Jyoti B. , Paranjape T. , Pathak S. , Tiwari S. , and Bedi N. S. , Comparison of Pretreatment by Different Analgesics on Post-Operative Endodontic Pain: A Clinical Study, Journal of International Oral Health. (2016) 8, no. 1, 10.4103/0976-7428.199494, 109.

[16] Attar S. , Bowles W. R. , Baisden M. K. , Hodges J. S. , and McClanahan S. B. , Evaluation of Pretreatment Analgesia and Endodontic Treatment for Postoperative Endodontic Pain, Journal of Endodontics. (2008) 34, no. 6, 652–655, 10.1016/j.joen.2008.02.017. 18498882

[17] Benyamin R. , Trescot A. M. , and Datta S. , et al.Opioid Complications and Side Effects, Pain Physician. (2008) 11, no. 2 Suppl. 18443635

[18] Berthold R. C. B. , Uso de Anti-Inflamatórios na Exodontia de Terceiros Molares Retidos: Revisão Sistemática e Meta-análise, 2018, Tese (Doutorado em Odontologia)—Escola de Ciências da Saúde, Pontifícia Universidade Católica do Rio Grande do Sul, Porto Alegre Acessed: 29 jan. 2021 http://tede2.pucrs.br/tede2/handle/tede/8357.

[19] Pochapski M. T. , Santos F. A. , de Andrade E. D. , and Sydney G. B. , Effect of Pretreatment Dexamethasone on Postendodontic Pain, Oral Surgery, Oral Medicine, Oral Pathology, Oral Radiology, and Endodontology. (2009) 108, no. 5, 790–795, 10.1016/j.tripleo.2009.05.014. 19748294

[20] Konagala R. K. , Mandava J. , Pabbati R. K. , Anupreeta A. , Borugadda R. , and Ravi R. , Effect of Pretreatment Medication on Postendodontic Pain: A Double-Blind, Placebo-Controlled Study, Journal of Conservative Dentistry. (2019) 22, no. 1, 54–58. 30820083 10.4103/JCD.JCD_135_18 PMC6385568

[21] Doleman B. , Leonardi-Bee J. , and Heinink T. P. , et al.Pre-Emptive and Preventive NSAIDs for Postoperative Pain in Adults Undergoing All Types of Surgery, Cochrane Database of Systematic Reviews. (2021) 2021, no. 6, 10.1002/14651858.CD012978.pub2. PMC8203105 34125958

[22] Teja K. V. , Ramesh S. , and Vasundhara A. , Analgesic Effect of Preemptive Oral NSAIDs on Post-Endodontic Pain Levels in Single Visit Endodontics—A Systematic Review, Cumhuriyet Dental Journal. (2021) 24, no. 3, 286–298, 10.7126/cumudj.871091.

[23] Shamszadeh S. , Shirvani A. , Eghbal M. J. , and Asgary S. , Efficacy of Corticosteroids on Postoperative Endodontic Pain: A Systematic Review and Meta-Analysis, Journal of Endodontics. (2018) 44, no. 7, 1057–1065, 10.1016/j.joen.2018.03.010. 29709296

[24] Higgins J. P. T. , Thomas J. , and Chandler J. , et al.Cochrane Handbook for Systematic Reviews of Interventions Version 6.5 (updated August 2024), 2024, Cochrane.

[25] Pimenta R. P. , Takahashi C. M. , and Barberato-Filho S. , et al.Preemptive use of Anti-Inflammatories and Analgesics in Oral Surgery: A Review of Systematic Reviews, Frontiers in Pharmacology. (2024) 14, 10.3389/fphar.2023.1303382, 1303382. 38328575 PMC10847331

[26] Shea B. J. , Reeves B. C. , and Wells G. , et al.AMSTAR. 2: A Critical Appraisal Tool for Systematic Reviews That Include Randomised or Non-Randomised Studies of Healthcare Interventions, or Both, BMJ. (2017) 358, 10.1136/bmj.j4008, j4008. 28935701 PMC5833365

[27] Higgins J. P. T. and Green S. , Cochrane Handbook for Systematic Reviews of Interventions. Version 5.10, 2011, The Cochrane Collaboration.

[28] Guyatt G. H. , Thorlund K. , and Oxman A. D. , et al.GRADE Guidelines: 13. Preparing Summary of Findings Tables and Evidence Profiles—Continuous Outcomes, Journal of Clinical Epidemiology. (2013) 66, no. 2, 173–183, 10.1016/j.jclinepi.2012.08.001. 23116689

[29] Pieper D. , Antoine S. L. , Mathes T. , Neugebauer E. A. , and Eikermann M. , Systematic Review Finds Overlapping Reviews Were Not Mentioned in Every Other Overview, Journal of Clinical Epidemiology. (2014) 67, no. 4, 368–375. 24581293 10.1016/j.jclinepi.2013.11.007

[30] Aminoshariae A. , Kulild J. C. , Donaldson M. , and Hersh E. V. , Evidence-Based Recommendations for Analgesic Efficacy to Treat Pain of Endodontic Origin: A Systematic Review of Randomized Controlled Trials, The Journal of the American Dental Association. (2016) 147, no. 10, 826–839, 10.1016/j.adaj.2016.05.010. 27475974

[31] De Geus J. L. , Wambier L. M. , Boing T. F. , Loguercio A. D. , and Reis A. , Effects of Ibuprofen Compared to Other Premedication Drugs on the Risk and Intensity of Postendodontic Pain: A Systematic Review, European Endodontic Journal. (2018) 3, no. 3, 123–133, 10.14744/eej.2018.83803. 32161868 PMC7006579

[32] Iranmanesh F. , Parirokh M. , Haghdoost A. A. , and Abbott P. V. , Effect of Corticosteroids on Pain Relief Following Root Canal Treatment: A Systematic Review, Iranian Endodontic Journal. (2017) 12, no. 2, 123–130. 28496516 10.22037/iej.2017.26 PMC5421265

[33] Jose J. , Teja K. V. , Palanivelu A. , Khandelwal A. , and Siddique R. , Analgesic Efficacy of Corticosteroids and Nonsteroidal Anti-Inflammatory Drugs Through Oral Route in the Reduction of Postendodontic Pain: A Systematic Review, Journal of Conservative Dentistry. (2022) 25, no. 1, 9–19, 10.4103/jcd.jcd_30_21. 35722072 PMC9200178

[34] Kumar G. , Sangwan P. , and Tewari S. , Effect of Premedication on Postoperative Pain After Root Canal Therapy in Patients With Irreversible Pulpitis: A Systematic Review and Meta-Analysis, Journal of Dental Anesthesia and Pain Medicine. (2021) 21, no. 5, 397–411, 10.17245/jdapm.2021.21.5.397. 34703890 PMC8520836

[35] Nagendrababu V. , Pulikkotil S. J. , Jinatongthai P. , Veettil S. K. , Teerawattanapong N. , and Gutmann J. L. , Efficacy and Safety of Oral Premedication on Pain After Nonsurgical Root Canal Treatment: A Systematic Review and Network Meta-Analysis of Randomized Controlled Trials, Journal of Endodontics. (2019) 45, no. 4, 364–371, 10.1016/j.joen.2018.10.016. 30737050

[36] Nath R. , Daneshmand A. , Sizemore D. , Guo J. , and Enciso R. , Efficacy of Corticosteroids for Postoperative Endodontic Pain: A Systematic Review and Meta-Analysis, Journal of Dental Anesthesia and Pain Medicine. (2018) 18, no. 4, 205–221, 10.17245/jdapm.2018.18.4.205. 30186968 PMC6115367

[37] Santini M. , Da Rosa R. A. , and Ferreira M. B. , et al.Medications Used for Prevention and Treatment of Postoperative Endodontic Pain: A Systematic Review, European Endodontic Journal. (2021) 6, no. 1, 15–24, 10.14744/eej.2020.85856. 33609020 PMC8056801

[38] Shirvani A. , Shamszadeh S. , Eghbal M. J. , and Asgary S. , The Efficacy of Non-Narcotic Analgesics on Post-Operative Endodontic Pain: A Systematic Review and Meta-Analysis, Journal of Oral Rehabilitation. (2017) 44, no. 9, 709–721, 10.1111/joor.12519. 28449307

[39] Smith E. A. , Marshall J. G. , Selph S. S. , Barker D. R. , and Sedgley C. M. , Nonsteroidal Anti-Inflammatory Drugs for Managing Postoperative Endodontic Pain in Patients Who Present With Preoperative Pain: A Systematic Review and Meta-Analysis, Journal of Endodontics. (2017) 43, no. 1, 7–15, 10.1016/j.joen.2016.09.010. 27939729

[40] Suneelkumar C. , Subha A. , and Gogala D. , Effect of Preoperative Corticosteroids in Patients With Symptomatic Pulpitis on Postoperative Pain After Single-Visit Root Canal Treatment: A Systematic Review and Meta-Analysis, Journal of Endodontics. (2018) 44, no. 9, 1347–1354, 10.1016/j.joen.2018.05.015. 30054100

[41] Mehrvarzfar P. , Esnashari E. , Salmanzadeh R. , Fazlyab M. , and Fazlyab M. , Effect of Dexamethasone Intraligamentary Injection on Post-Endodontic Pain in Patients With Symptomatic Irreversible Pulpitis: A Randomized Controlled Clinical Trial, Iranian Endodontic Journal. (2016) 11, no. 4, 261–266, 10.22037/iej.2016.2. 27790253 PMC5069900

[42] Mello P. S. , Analgesia Preemptiva com Dexametasona ou Ibuprofeno em Tratamentos e Retratamentos Endodônticos com Ampliação Foraminal, Dissertação—Faculdade de Odontologia de Piracicaba, 2014, Universidade Estadual de Campinas.

[43] Sharma N. , Nikhil V. , and Gupta S. , Effect of Preoperative Administration of Steroid with Different Routes on Post Endodontic Pain: A Randomized Placebo Controlled Clinical Trial, Endodontology. (2015) 27, no. 2, 107–112, 10.4103/0970-7212.218373.

[44] Jalalzadeh S. M. , Mamavi A. , Shahriari S. , Santos F. A. , and Pochapski M. T. , Effect of Pretreatment Prednisolone on Postendodontic Pain: A Double-Blind Parallel-Randomized Clinical Trial, Journal of Endodontics. (2010) 36, no. 6, 978–981, 10.1016/j.joen.2010.03.015. 20478449

[45] Kaufman E. , Heling I. , and Rotstein I. , et al.Intraligamentary Injection of Slow-Release Methylprednisolone for the Prevention of Pain After Endodontic Treatment, Oral Surgery, Oral Medicine, Oral Pathology. (1994) 77, no. 6, 651–654, 10.1016/0030-4220(94)90329-8. 8065733

[46] Arslan H. , Topcuoglu H. S. , and Aladag H. , Effectiveness of Tenoxicam and Ibuprofen for Pain Prevention Following Endodontic Therapy in Comparison to Placebo: A Randomized Double-Blind Clinical Trial, Journal of Oral Science. (2011) 53, no. 2, 157–161, 10.2334/josnusd.53.157. 21712619

[47] Dejkam S. M. , Kashefienejad M. , Mirzaeerad S. , Mghadamnia A. A. , and Gholinia H. , Evaluation of Pretreatment With Gelofen and Novafen on Pain Relief After Endodontic Treatment: A Double-Blind Randomized Clinical Trial Study, Caspian Journal of Dental Research. (2015) 4, 37–42.

[48] Ehsani M. , Moghadamnia A. A. , and Zahedpasha S. , et al.The Role of Prophylactic Ibuprofen and N-Acetylcysteine on the Level of Cytokines in Periapical Exudates and the Post-Treatment Pain, DARU Journal of Pharmaceutical Sciences. (2012) 20, no. 1, 10.1186/2008-2231-20-30, 30. 23351387 PMC3555796

[49] Khorasani M. M. Y. and Mahmud M. , Efficacy of Prophylactic use of Sulindac in Comparison With Ibuprofen on Post-Operative Endodontic Pain, Journal of Mashhad Dental School. (2011) 35, no. 4, 315–324.

[50] Menke E. R. , Jackson C. R. , Bagby M. D. , and Tracy T. S. , The Effectiveness of Prophylactic Etodolac on Postendodontic Pain, Journal of Endodontics. (2000) 26, no. 12, 712–715, 10.1097/00004770-200012000-00010. 11471639

[51] Mirzaie M. , Kavosi A. , Atbaie A. , Moazami F. , and Nooribaiat S. , Effect of Premedication With Celecoxib and Gelofen on Reduction of Post-Endodontic Pain, Journal of Dental Medicine. (2011) 24, no. 2, 172–180.

[52] Mokhtari F. , Yazdi K. , Mahabadi A. M. , Modaresi S. J. , and Hamzeheil Z. , Effect of Premedication With Indomethacin and Ibuprofen on Postoperative Endodontic Pain: A Clinical Trial, Iranian Endodontic Journal. (2016) 11, no. 1, 57–62, 10.7508/iej.2016.01.011. 26843879 PMC4731535

[53] Ramazani M. , Hamidi M. R. , Moghaddamnia A. A. , Ramazani N. , and Zarenejad N. , The Prophylactic Effects of Zintoma and Ibuprofen on Post-Endodontic Pain of Molars With Irreversible Pulpitis: A Randomized Clinical Trial, Iranian Endodontic Journal. (2013) 8, no. 3, 129–134, 10.5505/agri.2013.55477. 23922575 PMC3734516

[54] Saatchi M. , Mosavat F. , Razmara F. , and Soleymani B. , Comparison of the Effect of Ibuprofen and Slow-Released Diclofenac Sodium in Controlling Post-Endodontic Pain, Journal of Dental Medicine. (2010) 22, no. 4, 185–191.

[55] Akhlaghi N. , Azarshab M. , Akhoundi N. , and Meraji N. , The Effect of Ketorolac Buccal Infiltration on Postoperative Endodontic Pain: A Prospective, Double-Blind, Randomized, Controlled Clinical Trial, Quintessence International. (2019) 50, no. 7, 540–546, 10.3290/j.qi.a42654. 31187102

[56] Mellor A. C. , Dorman M. L. , and Girdler N. M. , The use of an Intra-Oral Injection of Ketorolac in the Treatment of Irreversible Pulpitis, International Endodontic Journal. (2005) 38, no. 11, 789–792, 10.1111/j.1365-2591.2005.01015_1.x. 16218969

[57] Penniston S. G. and Hargreaves K. M. , Evaluation of Periapical Injection of Ketorolac for Management of Endodontic Pain, Journal of Endodontics. (1996) 22, no. 2, 55–59, 10.1016/S0099-2399(96)80272-X. 8935018

[58] Sethi P. , Agarwal M. , Chourasia H. R. , and Singh M. P. , Effect of Single Dose Pretreatment Analgesia With Three Different Analgesics on Postoperative Endodontic Pain: A Randomized Clinical Trial, Journal of Conservative Dentistry. (2014) 17, no. 6, 517–521, 10.4103/0972-0707.144574. 25506136 PMC4252922

[59] Jenarthanan S. and Subbarao C. , Comparative Evaluation of the Efficacy of Diclofenac Sodium Administered Using Different Delivery Routes in the Management of Endodontic Pain: A Randomized Controlled Clinical Trial, Journal of Conservative Dentistry. (2018) 21, no. 3, 297–301, 10.4103/JCD.JCD_140_17. 29899633 PMC5977779

[60] Metri M. , Hegde S. , and Bhandi S. , Effect of Pretreatment Diclofenac Sodium on Postendodontic Pain: A Randomized Controlled Trial, Journal of Conservative Dentistry. (2016) 19, no. 1, 7–10, 10.4103/0972-0707.173183. 26957785 PMC4760019

[61] Rashka B. , Ganesh B. , Aditya S. , Shruti B. , and Mithra N. H. , Evaluation of the Efficiency of Intraligamentary Diclofenac Sodium in Reducing Postoperative Endodontic Pain in Patients With Irreversible Pulpitis, International Research Journal of Pharmacy. (2013) 4, no. 3, 237–239, 10.7897/2230-8407.04351.

[62] Atbaei A. and Mortazavi N. , Prophylactic Intraligamentary Injection of Piroxicam (feldene) for the Management of Post-Endodontic Pain in Molar Teeth With Irreversible Pulpitis, Australian Endodontic Journal. (2012) 38, no. 1, 31–35, 10.1111/j.1747-4477.2010.00274.x. 22432824

[63] Joshi N. , Mathew S. , George J. V. , Hegde S. , Bhandi S. , and Madhu K. S. , Comparative Evaluation of the Efficacy of Two Modes of Delivery of Piroxicam (Dolonex) for the Management of Postendodontic Pain: A Randomized Control Trial, Journal of Conservative Dentistry. (2016) 19, no. 4, 301–305, 10.4103/0972-0707.186454. 27563175 PMC4979273

[64] Ashraf H. , Naghlachi T. , and Ketabi M. A. , Comparison of the Efficacy of Prophylactic Celecoxib, a Cox-2 Inhibitor Made in Iran, and Placebo for Post-Endodontic Pain Reduction: A Clinical Double-Blind Study, Journal of Dental School-Shahid Beheshti University of Medical Sciences. (2013) 31, no. 2, 82–88.

[65] Flath R. K. , Hicks M. L. , Dionne R. A. , and Pelleu G. B.Jr., Pain Suppression After Pulpectomy With Preoperative Flurbiprofen, Journal of Endodontics. (1987) 13, no. 7, 339–347, 10.1016/S0099-2399(87)80116-4. 3327906

[66] Işik B. , Yaman S. , Aktuna S. , and Turan A. , Analgesic Efficacy of Prophylactic Gabapentin and Lornoxicam in Preventing Postendodontic Pain, Pain Medicine. (2014) 15, no. 12, 2150–2155, 10.1111/pme.12536. 25138784

