# Supplementary material for: Effectiveness and Safety of Pretreatment Analgesics and Anti‐Inflammatory Drugs in Endodontic Pain Management: Overview of Systematic Reviews
Source: Int J Dent. 2026 Aug 9;2026:7258438. doi: 10.1155/ijod/7258438 (PMC13454743; doi:10.1155/ijod/7258438)
Supplement: Supplementary file 1 [file IJOD-2026-7258438-s001.docx]

**Supporting Information 1:** Search strategies

| **#1**  **Dental procedures** | (Maxillofacial Surgery) OR (Oral Surgery) OR Exodontics OR (Third Molar) OR (Third Molars) OR (Wisdom Tooth) OR (Wisdom Teeth) OR (Impacted Tooth) OR (Impacted Teeth) OR (Dental Implant) OR (Dental Implants) OR (Unerupted Tooth) OR (Unerupted Teeth) OR (Supernumerary Tooth) OR (Supernumerary Teeth) OR (Fourth Molar) OR (Fourth Molars) OR (Bone Transplantation) OR (Bone Grafting) OR Autograft OR (Autologous Transplants) OR (Autologous Transplant) OR Autotransplants OR Autotransplant OR Allografts OR Allograft OR (Allogeneic Transplants) OR (Allogeneic Transplant) OR (Allogeneic Grafts) OR (Allogeneic Graft) OR Homografts OR Homograft OR (Homologous Transplants) OR (Homologous Transplant) OR Heterografts OR Heterograft OR Xenografts OR Xenograft OR Pulpitis OR Pulpitides OR (Endodontic Inflammation) OR (Endodontic Inflammations) OR endodontics OR endodontology OR (Dental Pulp Diseases) OR (Dental Pulp Disease) OR (root canal therapy) OR (root canal therapies) OR endodontic OR (periapical periodontitis) OR (Periapical Periodontitides) OR (Apical Periodontitides) OR (Apical Periodontitis) OR (Acute Nonsuppurative Periodontitides) OR (Acute Nonsuppurative Periodontitis) OR (dental pulp necrosis) OR (Dental Pulp Necroses) OR (Dental Pulp Necrosis) OR (Pulp Necroses) OR (Pulp Necrosis) OR (dental pulp gangrene) OR (dental pulp gangrenes) OR (Pulp Gangrene) OR (Pulp Gangrenes) OR (Pulp Mummification) OR (Pulp Mummifications) OR (Dental Pulp Autolysis) OR (Dental Pulp Autolyses) OR (nonvital tooth) OR (tooth devitalized) OR (tooth pulpless) OR (teeth pulpless) OR (teeth devitalized) OR (nonvital teeth) OR (teeth endodontically-treated) OR (teeth endodontically treated) OR (tooth endodontically-treated) OR (tooth endodontically treated) OR (granuloma periapical) OR (granulomas periapical) OR (periapical periodontitis chronic nonsuppurative) OR (periodontitis apical chronic nonsuppurative) OR (dental granulomas) OR (dental granuloma) OR (periodontitis apical suppurative) OR (abscess periapical) OR (abscesses periapical) OR (periapical periodontitides suppurative) OR (alveolar abscess apical) OR (abscesses apical alveolar) OR (dentoalveolar abscess apical) OR (abscesses apical dentoalveolar) OR Pulpectomy OR Pulpectomies OR (root canal preparation) OR (root canal preparations) |
| --- | --- |
| **#2 Drugs** | (Adrenal Cortex Hormones) OR Corticosteroids OR Corticosteroid OR Corticosteroids OR Corticosteroid OR (Glucocorticosteroids) OR Glucocorticosteroid OR (Glucocorticosteroid Effect) OR (Glucorticosteroid Effects) OR (Anti-Inflammatory Agents) OR (Anti Inflammatory Agents) OR (Antiinflammatory Agent) OR (Antiinflammatory Agents) OR Anti-Inflammatories OR (Anti Inflammatories) OR (Antiinflammatories) OR (Anti-Inflammatory Agent) OR (Anti Inflammatory Agent) OR (Agents Anti-Inflammatory) OR (Agents Anti Inflammatory) OR Dexamethasone OR Methylfluorprednisolone OR Betamethasone OR Flubenisolone OR Betadexamethasone OR (Methylprednisolone Acetate) OR Methylprednisolone OR Methylprednisolone-21-acetate OR (Methylprednisolone 21 acetate) OR Acetyl-Methylprednisolone OR (Acetyl Methylprednisolone) OR 6-Methylprednisolone OR (6 Methylprednisolone) OR [Prednisone](https://www.ncbi.nlm.nih.gov/mesh/68011241) OR Dehydrocortisone OR delta-Cortisone OR Prednisolone OR Hidrocortisone OR cortisol OR (Anti-Inflammatory Agents Non-Steroidal) OR NSAID OR (Nonsteroidal Anti-Inflammatory Agent) OR (Agent Nonsteroidal Anti-Inflammatory) OR (Anti-Inflammatory Agent Nonsteroidal) OR (Nonsteroidal Anti Inflammatory Agent) OR NSAIDS OR (Antiinflammatory Agents Non Steroidal) OR (Antiinflammatory Agents Nonsteroidal) OR (Nonsteroidal Antiinflammatory Agents) OR (Non-Steroidal Anti-Inflammatory Agents) OR (Non Steroidal Anti Inflammatory Agents) OR (Nonsteroidal Anti-Inflammatory Agents) OR (Nonsteroidal Anti Inflammatory Agents) OR (Non-Steroidal Anti-Inflammatory Agent) OR (Agent Non-Steroidal Anti-Inflammatory) OR (Anti-Inflammatory Agent Non-Steroidal) OR (Non Steroidal Anti Inflammatory Agent) OR (Anti Inflammatory Agents Nonsteroidal) OR (Analgesics Anti-Inflammatory) OR (Anti-Inflammatory Analgesics) OR (Aspirin-Like Agents) OR (Aspirin Like Agents) OR (Aspirin-Like Agent) OR (Agent Aspirin-Like) OR (Aspirin Like Agent) OR ([Cyclooxygenase 2 Inhibitors](https://www.ncbi.nlm.nih.gov/mesh/68052246)) OR (Cyclooxygenase 2 Inhibitor) OR (COX-2 Inhibitor) OR (COX 2 Inhibitor) OR Coxib OR Coxibs OR (COX-2 Inhibitors) OR (COX 2 Inhibitors) OR (COX2 Inhibitors) OR (COX2 Inhibitor) OR (Cyclooxygenase-2 Inhibitors) OR (Cyclooxygenase-2 Inhibitor) OR Celecoxib OR Ketoprofen OR diclofenac OR flurbiprofen OR Ibuprofen OR Indomethacin OR ketorolac OR lornoxicam OR (mefenamic acid) OR meloxicam OR naproxen OR piroxicam OR sulindac OR celecoxib OR valdecoxib OR lumiracoxib OR rofecoxib OR etoricoxib OR parecoxib OR etodolac OR Analgesics OR Anodynes OR (Analgesic Drugs) OR Analgesic OR (Analgesic Agents) OR (Antinociceptive Agents) OR (Agent Antinociceptive) OR Dipyrone OR Methamizole OR Metamizol OR Dipyronium OR Metamizole OR Acetaminophen OR Acetominophen OR Acetaminophen OR Acetamidophenol OR (Analgesics Opioid) OR (Opioid Analgesics) OR (Opioid Analgesic) OR Opioids OR Opioid OR (Opioid Partial Agonists) OR (Agonists Opioid Partial) OR (Full Opioid Agonists) OR tramadol OR codeine OR pentazocine OR tapentadol OR oxycodone |
| **#3**  **Preventive use** | Preemptive OR Preemptive OR (Pre emptive) OR Preventive OR Premedication OR (Preoperative Care) OR (Care Preoperative) OR (Preoperative Procedure) OR (Procedures Preoperative) OR (Preoperative Period) OR (Period Preoperative) OR (Acute pain) OR (Acute pains) OR (Post-surgical Pain) OR (Pain Post-surgical) OR (Post surgical Pain) OR (Pain Post-operative) OR (Pain Post operative) OR (Postsurgical Pain) OR (Post-operative Pain) OR (Post operative Pain) OR (Post-operative Pains) OR (Postoperative Pain) OR (Postoperative Pain Chronic) OR (Pain Chronic Postoperative) OR (Chronic Postoperative Pain) OR (Chronic Post-surgical Pain) OR (Chronic Post surgical Pain) OR (Pain Chronic Post-surgical) OR (Post-surgical Pain Chronic) OR (Chronic Postsurgical Pain) OR (Chronic Postsurgical Pains) OR (Pain Chronic Postsurgical) OR (Postsurgical Pain Chronic) OR (Persistent Postsurgical Pain) OR (Pain Persistent Postsurgical) OR (Postsurgical Pain Persistent) OR (Post-operative Pain Chronic) OR (Pain Chronic Post-operative) OR (Post operative Pain Chronic) OR (Chronic Post-operative Pain) OR (Chronic Post operative Pain) OR (Postoperative Pain Acute) OR (Pain Acute Postoperative) OR (Acute Postoperative Pain) OR (Acute Post-operative Pain) OR (Acute Post operative Pain) OR (Post-operative Pain Acute) OR (Pain Acute Post-operative) OR (Post operative Pain Acute) |
| **#4**  **Systematic review** | (((systematic review[ti] OR systematic literature review[ti] OR systematic scoping review[ti] OR systematic narrative review[ti] OR systematic qualitative review[ti] OR systematic evidence review[ti] OR systematic quantitative review[ti] OR systematic meta-review[ti] OR systematic critical review[ti] OR systematic mixed studies review[ti] OR systematic mapping review[ti] OR systematic cochrane review[ti] OR systematic search and review[ti] OR systematic integrative review[ti]) NOT comment[pt] NOT (protocol[ti] OR protocols[ti])) NOT MEDLINE [subset]) OR (Cochrane Database Syst Rev[ta] AND review[pt]) OR systematic review[pt] |
| **#5** | **#1 AND #2 AND #3 AND #4** |

**MEDLINE (PubMed) (n=362 results)**

 ((((Pulpitis OR Pulpitides OR (Endodontic Inflammation) OR (Endodontic Inflammations) OR endodontics OR endodontology OR (Dental Pulp Diseases) OR (Dental Pulp Disease) OR (root canal therapy) OR (root canal therapies) OR endodontic OR (periapical periodontitis) OR (Periapical Periodontitides) OR (Apical Periodontitides) OR (Apical Periodontitis) OR (Acute Nonsuppurative Periodontitides) OR (Acute Nonsuppurative Periodontitis) OR (dental pulp necrosis) OR (Dental Pulp Necroses) OR (Dental Pulp Necrosis) OR (Pulp Necroses) OR (Pulp Necrosis) OR (dental pulp gangrene) OR (dental pulp gangrenes) OR (Pulp Gangrene) OR (Pulp Gangrenes) OR (Pulp Mummification) OR (Pulp Mummifications) OR (Dental Pulp Autolysis) OR (Dental Pulp Autolyses) OR (nonvital tooth) OR (tooth devitalized) OR (tooth pulpless) OR (teeth pulpless) OR (teeth devitalized) OR (nonvital teeth) OR (teeth endodontically-treated) OR (teeth endodontically treated) OR (tooth endodontically-treated) OR (tooth endodontically treated) OR (granuloma periapical) OR (granulomas periapical) OR (periapical periodontitis chronic nonsuppurative) OR (periodontitis apical chronic nonsuppurative) OR (dental granulomas) OR (dental granuloma) OR (periodontitis apical suppurative) OR (abscess periapical) OR (abscesses periapical) OR (periapical periodontitides suppurative) OR (alveolar abscess apical) OR (abscesses apical alveolar) OR (dentoalveolar abscess apical) OR (abscesses apical dentoalveolar) OR Pulpectomy OR Pulpectomies OR (root canal preparation) OR (root canal preparations)) OR ((Maxillofacial Surgery) OR (Oral Surgery) OR Exodontics OR (Third Molar) OR (Third Molars) OR (Wisdom Tooth) OR (Wisdom Teeth) OR (Impacted Tooth) OR (Impacted Teeth) OR (Dental Implant) OR (Dental Implants) OR (Unerupted Tooth) OR (Unerupted Teeth) OR (Supernumerary Tooth) OR (Supernumerary Teeth) OR (Fourth Molar) OR (Fourth Molars) OR (Bone Transplantation) OR (Bone Grafting) OR Autograft OR (Autologous Transplants) OR (Autologous Transplant) OR Autotransplants OR Autotransplant OR Allografts OR Allograft OR (Allogeneic Transplants) OR (Allogeneic Transplant) OR (Allogeneic Grafts) OR (Allogeneic Graft) OR Homografts OR Homograft OR (Homologous Transplants) OR (Homologous Transplant) OR Heterografts OR Heterograft OR Xenografts OR Xenograft)) AND ((Adrenal Cortex Hormones) OR Corticosteroids OR Corticosteroid OR Corticoids OR Corticoid OR (Glucocorticoids) OR Glucocorticoid OR (Glucocorticoid Effect) OR (Glucorticoid Effects) OR (Anti-Inflammatory Agents) OR (Anti Inflammatory Agents) OR (Antiinflammatory Agent) OR (Antiinflammatory Agents) OR Anti-Inflammatories OR (Anti Inflammatories) OR (Antiinflammatories) OR (Anti-Inflammatory Agent) OR (Anti Inflammatory Agent) OR (Agents Anti-Inflammatory) OR (Agents Anti Inflammatory) OR Dexamethasone OR Methylfluorprednisolone OR Betamethasone OR Flubenisolone OR Betadexamethasone OR (Methylprednisolone Acetate) OR Methylprednisolone OR Methylprednisolone-21-acetate OR (Methylprednisolone 21 acetate) OR Acetyl-Methylprednisolone OR (Acetyl Methylprednisolone) OR 6-Methylprednisolone OR (6 Methylprednisolone) OR Prednisone OR Dehydrocortisone OR delta-Cortisone OR Prednisolone OR Hidrocortisone OR cortisol OR (Anti-Inflammatory Agents Non-Steroidal) OR NSAID OR (Nonsteroidal Anti-Inflammatory Agent) OR (Agent Nonsteroidal Anti-Inflammatory) OR (Anti-Inflammatory Agent Nonsteroidal) OR (Nonsteroidal Anti Inflammatory Agent) OR NSAIDS OR (Antiinflammatory Agents Non Steroidal) OR (Antiinflammatory Agents Nonsteroidal) OR (Nonsteroidal Antiinflammatory Agents) OR (Non-Steroidal Anti-Inflammatory Agents) OR (Non Steroidal Anti Inflammatory Agents) OR (Nonsteroidal Anti-Inflammatory Agents) OR (Nonsteroidal Anti Inflammatory Agents) OR (Non-Steroidal Anti-Inflammatory Agent) OR (Agent Non-Steroidal Anti-Inflammatory) OR (Anti-Inflammatory Agent Non-Steroidal) OR (Non Steroidal Anti Inflammatory Agent) OR (Anti Inflammatory Agents Nonsteroidal) OR (Analgesics Anti-Inflammatory) OR (Anti-Inflammatory Analgesics) OR (Aspirin-Like Agents) OR (Aspirin Like Agents) OR (Aspirin-Like Agent) OR (Agent Aspirin-Like) OR (Aspirin Like Agent) OR (Cyclooxygenase 2 Inhibitors) OR (Cyclooxygenase 2 Inhibitor) OR (COX-2 Inhibitor) OR (COX 2 Inhibitor) OR Coxib OR Coxibs OR (COX-2 Inhibitors) OR (COX 2 Inhibitors) OR (COX2 Inhibitors) OR (COX2 Inhibitor) OR (Cyclooxygenase-2 Inhibitors) OR (Cyclooxygenase-2 Inhibitor) OR Celecoxib OR Ketoprofen OR diclofenac OR flurbiprofen OR Ibuprofen OR Indomethacin OR ketorolac OR lornoxicam OR (mefenamic acid) OR meloxicam OR naproxen OR piroxicam OR sulindac OR celecoxib OR valdecoxib OR lumiracoxib OR rofecoxib OR etoricoxib OR parecoxib OR etodolac OR Analgesics OR Anodynes OR (Analgesic Drugs) OR Analgesic OR (Analgesic Agents) OR (Antinociceptive Agents) OR (Agent Antinociceptive) OR Dipyrone OR Methamizole OR Metamizol OR Dipyronium OR Metamizole OR Acetaminophen OR Acetominophen OR Acetaminophen OR Acetamidophenol OR (Analgesics Opioid) OR (Opioid Analgesics) OR (Opioid Analgesic) OR Opioids OR Opioid OR (Opioid Partial Agonists) OR (Agonists Opioid Partial) OR (Full Opioid Agonists) OR tramadol OR codeine OR pentazocine OR tapentadol OR oxycodone)) AND (Preemptive OR Preemptive OR (Pre emptive) OR Preventive OR Premedication OR (Preoperative Care) OR (Care Preoperative) OR (Preoperative Procedure) OR (Procedures Preoperative) OR (Preoperative Period) OR (Period Preoperative) OR (Acute pain) OR (Acute pains) OR (Post-surgical Pain) OR (Pain Post-surgical) OR (Post surgical Pain) OR (Pain Post-operative) OR (Pain Post operative) OR (Postsurgical Pain) OR (Post-operative Pain) OR (Post operative Pain) OR (Post-operative Pains) OR (Postoperative Pain) OR (Postoperative Pain Chronic) OR (Pain Chronic Postoperative) OR (Chronic Postoperative Pain) OR (Chronic Post-surgical Pain) OR (Chronic Post surgical Pain) OR (Pain Chronic Post-surgical) OR (Post-surgical Pain Chronic) OR (Chronic Postsurgical Pain) OR (Chronic Postsurgical Pains) OR (Pain Chronic Postsurgical) OR (Postsurgical Pain Chronic) OR (Persistent Postsurgical Pain) OR (Pain Persistent Postsurgical) OR (Postsurgical Pain Persistent) OR (Post-operative Pain Chronic) OR (Pain Chronic Post-operative) OR (Post operative Pain Chronic) OR (Chronic Post-operative Pain) OR (Chronic Post operative Pain) OR (Postoperative Pain Acute) OR (Pain Acute Postoperative) OR (Acute Postoperative Pain) OR (Acute Post-operative Pain) OR (Acute Post operative Pain) OR (Post-operative Pain Acute) OR (Pain Acute Post-operative) OR (Post operative Pain Acute))) AND ((((systematic review[ti] OR systematic literature review[ti] OR systematic scoping review[ti] OR systematic narrative review[ti] OR systematic qualitative review[ti] OR systematic evidence review[ti] OR systematic quantitative review[ti] OR systematic meta-review[ti] OR systematic critical review[ti] OR systematic mixed studies review[ti] OR systematic mapping review[ti] OR systematic cochrane review[ti] OR systematic search and review[ti] OR systematic integrative review[ti]) NOT comment[pt] NOT (protocol[ti] OR protocols[ti])) NOT MEDLINE [subset]) OR (Cochrane Database Syst Rev[ta] AND review[pt]) OR systematic review[pt]) Sort by: Most Recent

**EMBASE (n=304 results)**

Query('pulpitis'/exp OR pulpitis OR pulpitides OR (endodontic AND ('inflammation'/exp OR inflammation)) OR (endodontic AND inflammations) OR 'endodontics'/exp OR endodontics OR endodontology OR (('dental'/exp OR dental) AND ('pulp'/exp OR pulp) AND ('diseases'/exp OR diseases)) OR (('dental'/exp OR dental) AND ('pulp'/exp OR pulp) AND ('disease'/exp OR disease)) OR (('root'/exp OR root) AND canal AND ('therapy'/exp OR therapy)) OR (('root'/exp OR root) AND canal AND therapies) OR endodontic OR (periapical AND ('periodontitis'/exp OR periodontitis)) OR (periapical AND periodontitides) OR (apical AND periodontitides) OR (apical AND ('periodontitis'/exp OR periodontitis)) OR (acute AND nonsuppurative AND periodontitides) OR (acute AND nonsuppurative AND ('periodontitis'/exp OR periodontitis)) OR (('dental'/exp OR dental) AND ('pulp'/exp OR pulp) AND necroses) OR (('dental'/exp OR dental) AND ('pulp'/exp OR pulp) AND ('necrosis'/exp OR necrosis)) OR (('pulp'/exp OR pulp) AND necroses) OR (('pulp'/exp OR pulp) AND ('necrosis'/exp OR necrosis)) OR (('dental'/exp OR dental) AND ('pulp'/exp OR pulp) AND ('gangrene'/exp OR gangrene)) OR (('dental'/exp OR dental) AND ('pulp'/exp OR pulp) AND gangrenes) OR (('pulp'/exp OR pulp) AND ('gangrene'/exp OR gangrene)) OR (('pulp'/exp OR pulp) AND gangrenes) OR (('pulp'/exp OR pulp) AND ('mummification'/exp OR mummification)) OR (('pulp'/exp OR pulp) AND mummifications) OR (('dental'/exp OR dental) AND ('pulp'/exp OR pulp) AND ('autolysis'/exp OR autolysis)) OR (('dental'/exp OR dental) AND ('pulp'/exp OR pulp) AND autolyses) OR (nonvital AND ('tooth'/exp OR tooth)) OR (('tooth'/exp OR tooth) AND devitalized) OR (('tooth'/exp OR tooth) AND pulpless) OR (('teeth'/exp OR teeth) AND pulpless) OR (('teeth'/exp OR teeth) AND devitalized) OR (nonvital AND ('teeth'/exp OR teeth)) OR (('teeth'/exp OR teeth) AND 'endodontically treated') OR (('teeth'/exp OR teeth) AND endodontically AND treated) OR (('tooth'/exp OR tooth) AND 'endodontically treated') OR (('tooth'/exp OR tooth) AND endodontically AND treated) OR (('granuloma'/exp OR granuloma) AND periapical) OR (('granulomas'/exp OR granulomas) AND periapical) OR (periapical AND ('periodontitis'/exp OR periodontitis) AND chronic AND nonsuppurative) OR (('periodontitis'/exp OR periodontitis) AND apical AND chronic AND nonsuppurative) OR (('dental'/exp OR dental) AND ('granulomas'/exp OR granulomas)) OR (('dental'/exp OR dental) AND ('granuloma'/exp OR granuloma)) OR (('periodontitis'/exp OR periodontitis) AND apical AND suppurative) OR (('abscess'/exp OR abscess) AND periapical) OR (abscesses AND periapical) OR (periapical AND periodontitides AND suppurative) OR (alveolar AND ('abscess'/exp OR abscess) AND apical) OR (abscesses AND apical AND alveolar) OR (dentoalveolar AND ('abscess'/exp OR abscess) AND apical) OR (abscesses AND apical AND dentoalveolar) OR 'pulpectomy'/exp OR pulpectomy OR pulpectomies OR (('root'/exp OR root) AND canal AND ('preparation'/exp OR preparation)) OR (('root'/exp OR root) AND canal AND preparations) OR (maxillofacial AND surgery) OR (oral AND surgery) OR exodontics OR (third AND molar) OR (third AND molars) OR (wisdom AND tooth) OR (wisdom AND teeth) OR (impacted AND tooth) OR (impacted AND teeth) OR (dental AND implant) OR (dental AND implants) OR (unerupted AND tooth) OR (unerupted AND teeth) OR (supernumerary AND tooth) OR (supernumerary AND teeth) OR (fourth AND molar) OR (fourth AND molars) OR (bone AND transplantation) OR (bone AND grafting) OR autograft OR (autologous AND transplants) OR (autologous AND transplant) OR autotransplants OR autotransplant OR allografts OR allograft OR (allogeneic AND transplants) OR (allogeneic AND transplant) OR (allogeneic AND grafts) OR (allogeneic AND graft) OR homografts OR homograft OR (homologous AND transplants) OR (homologous AND transplant) OR heterografts OR heterograft OR xenografts OR xenograft) AND (adrenal AND cortex AND hormones OR corticosteroids OR corticosteroid OR corticoids OR corticoid OR glucocorticoids OR glucocorticoid OR (glucocorticoid AND effect) OR (glucorticoid AND effects) OR ('anti inflammatory' AND agents) OR (anti AND inflammatory AND agents) OR (antiinflammatory AND agent) OR (antiinflammatory AND agents) OR 'anti inflammatories' OR (anti AND inflammatories) OR antiinflammatories OR ('anti inflammatory' AND agent) OR (anti AND inflammatory AND agent) OR (agents AND 'anti inflammatory') OR (agents AND anti AND inflammatory) OR dexamethasone OR methylfluorprednisolone OR betamethasone OR flubenisolone OR betadexamethasone OR (methylprednisolone AND acetate) OR methylprednisolone OR 'methylprednisolone 21 acetate' OR (methylprednisolone AND 21 AND acetate) OR 'acetyl methylprednisolone' OR (acetyl AND methylprednisolone) OR '6 methylprednisolone' OR (6 AND methylprednisolone) OR prednisone OR dehydrocortisone OR 'delta cortisone' OR prednisolone OR hidrocortisone OR cortisol OR ('anti inflammatory' AND agents AND 'non steroidal') OR nsaid OR (nonsteroidal AND 'anti inflammatory' AND agent) OR (agent AND nonsteroidal AND 'anti inflammatory') OR ('anti inflammatory' AND agent AND nonsteroidal) OR (nonsteroidal AND anti AND inflammatory AND agent) OR NSAIDS OR (antiinflammatory AND agents AND non AND steroidal) OR (antiinflammatory AND agents AND nonsteroidal) OR (nonsteroidal AND antiinflammatory AND agents) OR ('non steroidal' AND 'anti inflammatory' AND agents) OR (non AND steroidal AND anti AND inflammatory AND agents) OR (nonsteroidal AND 'anti inflammatory' AND agents) OR (nonsteroidal AND anti AND inflammatory AND agents) OR ('non steroidal' AND 'anti inflammatory' AND agent) OR (agent AND 'non steroidal' AND 'anti inflammatory') OR ('anti inflammatory' AND agent AND 'non steroidal') OR (non AND steroidal AND anti AND inflammatory AND agent) OR (anti AND inflammatory AND agents AND nonsteroidal) OR (analgesics AND 'anti inflammatory') OR ('anti inflammatory' AND analgesics) OR ('aspirin like' AND agents) OR (aspirin AND like AND agents) OR ('aspirin like' AND agent) OR (agent AND 'aspirin like') OR (aspirin AND like AND agent) OR (cyclooxygenase AND 2 AND inhibitors) OR (cyclooxygenase AND 2 AND inhibitor) OR ('cox 2' AND inhibitor) OR (cox AND 2 AND inhibitor) OR coxib OR coxibs OR ('cox 2' AND inhibitors) OR (cox AND 2 AND inhibitors) OR (cox2 AND inhibitors) OR (cox2 AND inhibitor) OR ('cyclooxygenase 2' AND inhibitors) OR ('cyclooxygenase 2' AND inhibitor) OR ketoprofen OR diclofenac OR flurbiprofen OR ibuprofen OR indomethacin OR ketorolac OR lornoxicam OR (mefenamic AND acid) OR meloxicam OR naproxen OR piroxicam OR sulindac OR celecoxib OR valdecoxib OR lumiracoxib OR rofecoxib OR etoricoxib OR parecoxib OR etodolac OR analgesics OR anodynes OR (analgesic AND drugs) OR analgesic OR (analgesic AND agents) OR (antinociceptive AND agents) OR (agent AND antinociceptive) OR dipyrone OR methamizole OR metamizol OR dipyronium OR metamizole OR acetaminophen OR acetominophen OR acetaminophen OR acetamidophenol OR (analgesics AND opioid) OR (opioid AND analgesics) OR (opioid AND analgesic) OR opioids OR opioid OR (opioid AND partial AND agonists) OR (agonists AND opioid AND partial) OR (full AND opioid AND agonists) OR tramadol OR codeine OR pentazocine OR tapentadol OR oxycodone) AND (preemptive OR 'pre emptive' OR (pre AND emptive) OR preventive OR premedication OR (preoperative AND care) OR (care AND preoperative) OR (preoperative AND procedure) OR (procedures AND preoperative) OR (preoperative AND period) OR (period AND preoperative) OR (acute AND pain) OR (acute AND pains) OR ('post surgical' AND pain) OR (pain AND 'post surgical') OR (post AND surgical AND pain) OR (pain AND 'post operative') OR (pain AND post AND operative) OR (postsurgical AND pain) OR ('post operative' AND pain) OR (post AND operative AND pain) OR ('post operative' AND pains) OR (postoperative AND pain) OR (postoperative AND pain AND chronic) OR (pain AND chronic AND postoperative) OR (chronic AND postoperative AND pain) OR (chronic AND 'post surgical' AND pain) OR (chronic AND post AND surgical AND pain) OR (pain AND chronic AND 'post surgical') OR ('post surgical' AND pain AND chronic) OR (chronic AND postsurgical AND pain) OR (chronic AND postsurgical AND pains) OR (pain AND chronic AND postsurgical) OR (postsurgical AND pain AND chronic) OR (persistent AND postsurgical AND pain) OR (pain AND persistent AND postsurgical) OR (postsurgical AND pain AND persistent) OR ('post operative' AND pain AND chronic) OR (pain AND chronic AND 'post operative') OR (post AND operative AND pain AND chronic) OR (chronic AND 'post operative' AND pain) OR (chronic AND post AND operative AND pain) OR (postoperative AND pain AND acute) OR (pain AND acute AND postoperative) OR (acute AND postoperative AND pain) OR (acute AND 'post operative' AND pain) OR (acute AND post AND operative AND pain) OR ('post operative' AND pain AND acute) OR (pain AND acute AND 'post operative') OR (post AND operative AND pain AND acute)) AND ((('systematic review':ti OR 'systematic literature review':ti OR 'systematic scoping review':ti OR 'systematic narrative review':ti OR 'systematic qualitative review':ti OR 'systematic evidence review':ti OR 'systematic quantitative review':ti OR 'systematic meta-review':ti OR 'systematic critical review':ti OR 'systematic mixed studies review':ti OR 'systematic mapping review':ti OR 'systematic cochrane review':ti OR 'systematic search') AND review:ti OR 'systematic integrative review':ti) NOT comment:it NOT (protocol:ti OR protocols:ti) NOT 'medline [subset]' OR ('cochrane database syst rev[ta]' AND review:it) OR 'systematic review':it) AND [embase]/lim

**COCHRANE (n=73 results)**

#1= Pulpitis OR Pulpitides OR (Endodontic Inflammation) OR (Endodontic Inflammations) OR endodontics OR endodontology OR (Dental Pulp Diseases) OR (Dental Pulp Disease) OR (root canal therapy) OR (root canal therapies) OR endodontic OR (periapical periodontitis) OR (Periapical Periodontitides) OR (Apical Periodontitides) OR (Apical Periodontitis) OR (Acute Nonsuppurative Periodontitides) OR (Acute Nonsuppurative Periodontitis) OR (dental pulp necrosis) OR (Dental Pulp Necroses) OR (Dental Pulp Necrosis) OR (Pulp Necroses) OR (Pulp Necrosis) OR (dental pulp gangrene) OR (dental pulp gangrenes) OR (Pulp Gangrene) OR (Pulp Gangrenes) OR (Pulp Mummification) OR (Pulp Mummifications) OR (Dental Pulp Autolysis) OR (Dental Pulp Autolyses) OR (nonvital tooth) OR (tooth devitalized) OR (tooth pulpless) OR (teeth pulpless) OR (teeth devitalized) OR (nonvital teeth) OR (teeth endodontically-treated) OR (teeth endodontically treated) OR (tooth endodontically-treated) OR (tooth endodontically treated) OR (granuloma periapical) OR (granulomas periapical) OR (periapical periodontitis chronic nonsuppurative) OR (periodontitis apical chronic nonsuppurative) OR (dental granulomas) OR (dental granuloma) OR (periodontitis apical suppurative) OR (abscess periapical) OR (abscesses periapical) OR (periapical periodontitides suppurative) OR (alveolar abscess apical) OR (abscesses apical alveolar) OR (dentoalveolar abscess apical) OR (abscesses apical dentoalveolar) OR Pulpectomy OR Pulpectomies OR (root canal preparation) OR (root canal preparations)

#2= (Maxillofacial Surgery) OR (Oral Surgery) OR Exodontics OR (Third Molar) OR (Third Molars) OR (Wisdom Tooth) OR (Wisdom Teeth) OR (Impacted Tooth) OR (Impacted Teeth) OR (Dental Implant) OR (Dental Implants) OR (Unerupted Tooth) OR (Unerupted Teeth) OR (Supernumerary Tooth) OR (Supernumerary Teeth) OR (Fourth Molar) OR (Fourth Molars) OR (Bone Transplantation) OR (Bone Grafting) OR Autograft OR (Autologous Transplants) OR (Autologous Transplant) OR Autotransplants OR Autotransplant OR Allografts OR Allograft OR (Allogeneic Transplants) OR (Allogeneic Transplant) OR (Allogeneic Grafts) OR (Allogeneic Graft) OR Homografts OR Homograft OR (Homologous Transplants) OR (Homologous Transplant) OR Heterografts OR Heterograft OR Xenografts OR Xenograft

#3= (Adrenal Cortex Hormones) OR Corticosteroids OR Corticosteroid OR Corticoids OR Corticoid OR (Glucocorticoids) OR Glucocorticoid OR (Glucocorticoid Effect) OR (Glucorticoid Effects) OR (Anti-Inflammatory Agents) OR (Anti Inflammatory Agents) OR (Antiinflammatory Agent) OR (Antiinflammatory Agents) OR Anti-Inflammatories OR (Anti Inflammatories) OR (Antiinflammatories) OR (Anti-Inflammatory Agent) OR (Anti Inflammatory Agent) OR (Agents Anti-Inflammatory) OR (Agents Anti Inflammatory) OR Dexamethasone OR Methylfluorprednisolone OR Betamethasone OR Flubenisolone OR Betadexamethasone OR (Methylprednisolone Acetate) OR Methylprednisolone OR Acetyl-Methylprednisolone OR (Acetyl Methylprednisolone) OR Prednisone OR Dehydrocortisone OR delta-Cortisone OR Prednisolone OR Hidrocortisone OR cortisol OR (Anti-Inflammatory Agents Non-Steroidal) OR NSAID OR (Nonsteroidal Anti-Inflammatory Agent) OR (Agent Nonsteroidal Anti-Inflammatory) OR (Anti-Inflammatory Agent Nonsteroidal) OR (Nonsteroidal Anti Inflammatory Agent) OR NSAIDS OR (Antiinflammatory Agents Non Steroidal) OR (Antiinflammatory Agents Nonsteroidal) OR (Nonsteroidal Antiinflammatory Agents) OR (Non-Steroidal Anti-Inflammatory Agents) OR (Non Steroidal Anti Inflammatory Agents) OR (Nonsteroidal Anti-Inflammatory Agents) OR (Nonsteroidal Anti Inflammatory Agents) OR (Non-Steroidal Anti-Inflammatory Agent) OR (Agent Non-Steroidal Anti-Inflammatory) OR (Anti-Inflammatory Agent Non-Steroidal) OR (Non Steroidal Anti Inflammatory Agent) OR (Anti Inflammatory Agents Nonsteroidal) OR (Analgesics Anti-Inflammatory) OR (Anti-Inflammatory Analgesics) OR (Aspirin-Like Agents) OR (Aspirin Like Agents) OR (Aspirin-Like Agent) OR (Agent Aspirin-Like) OR (Aspirin Like Agent) OR (Cyclooxygenase 2 Inhibitors) OR (Cyclooxygenase 2 Inhibitor) OR (COX-2 Inhibitor) OR (COX 2 Inhibitor) OR Coxib OR Coxibs OR (COX-2 Inhibitors) OR (COX 2 Inhibitors) OR (COX2 Inhibitors) OR (COX2 Inhibitor) OR (Cyclooxygenase-2 Inhibitors) OR (Cyclooxygenase-2 Inhibitor) OR Celecoxib OR Ketoprofen OR diclofenac OR flurbiprofen OR Ibuprofen OR Indomethacin OR ketorolac OR lornoxicam OR (mefenamic acid) OR meloxicam OR naproxen OR piroxicam OR sulindac OR celecoxib OR valdecoxib OR lumiracoxib OR rofecoxib OR etoricoxib OR parecoxib OR etodolac OR Analgesics OR Anodynes OR (Analgesic Drugs) OR Analgesic OR (Analgesic Agents) OR (Antinociceptive Agents) OR (Agent Antinociceptive) OR Dipyrone OR Methamizole OR Metamizol OR Dipyronium OR Metamizole OR Acetaminophen OR Acetominophen OR Acetaminophen OR Acetamidophenol OR (Analgesics Opioid) OR (Opioid Analgesics) OR (Opioid Analgesic) OR Opioids OR Opioid OR (Opioid Partial Agonists) OR (Agonists Opioid Partial) OR (Full Opioid Agonists) OR tramadol OR codeine OR pentazocine OR tapentadol OR oxycodone

#4= Preemptive OR Preemptive OR (Pre emptive) OR Preventive OR Premedication OR (Preoperative Care) OR (Care Preoperative) OR (Preoperative Procedure) OR (Procedures Preoperative) OR (Preoperative Period) OR (Period Preoperative) OR (Acute pain) OR (Acute pains) OR (Post-surgical Pain) OR (Pain Post-surgical) OR (Post surgical Pain) OR (Pain Post-operative) OR (Pain Post operative) OR (Postsurgical Pain) OR (Post-operative Pain) OR (Post operative Pain) OR (Post-operative Pains) OR (Postoperative Pain) OR (Postoperative Pain Chronic) OR (Pain Chronic Postoperative) OR (Chronic Postoperative Pain) OR (Chronic Post-surgical Pain) OR (Chronic Post surgical Pain) OR (Pain Chronic Post-surgical) OR (Post-surgical Pain Chronic) OR (Chronic Postsurgical Pain) OR (Chronic Postsurgical Pains) OR (Pain Chronic Postsurgical) OR (Postsurgical Pain Chronic) OR (Persistent Postsurgical Pain) OR (Pain Persistent Postsurgical) OR (Postsurgical Pain Persistent) OR (Post-operative Pain Chronic) OR (Pain Chronic Post-operative) OR (Post operative Pain Chronic) OR (Chronic Post-operative Pain) OR (Chronic Post operative Pain) OR (Postoperative Pain Acute) OR (Pain Acute Postoperative) OR (Acute Postoperative Pain) OR (Acute Post-operative Pain) OR (Acute Post operative Pain) OR (Post-operative Pain Acute) OR (Pain Acute Post-operative) OR (Post operative Pain Acute)

#5= #1 OR #2

#6= #5 AND #4 AND #3 (n=501)

Only systematic reviews (n=63)

**WEB OF SCIENCE (n=218 results)**

#7= #6 AND #5 AND #4 AND #1 ([155](http://apps-webofknowledge.ez257.periodicos.capes.gov.br/summary.do?product=WOS&doc=1&qid=11&SID=7EGQ9RjqAZRCyhQcV4G&search_mode=CombineSearches&update_back2search_link_param=yes))

#6= #3 OR #2 ([453.911](http://apps-webofknowledge.ez257.periodicos.capes.gov.br/summary.do?product=WOS&doc=1&qid=10&SID=7EGQ9RjqAZRCyhQcV4G&search_mode=CombineSearches&update_back2search_link_param=yes))

#5= TS=(Preemptive OR Preemptive OR (Pre emptive) OR Preventive OR Premedication OR (Preoperative Care) OR (Care Preoperative) OR (Preoperative Procedure) OR (Procedures Preoperative) OR (Preoperative Period) OR (Period Preoperative) OR (Acute pain) OR (Acute pains) OR (Post-surgical Pain) OR (Pain Post-surgical) OR (Post surgical Pain) OR (Pain Post-operative) OR (Pain Post operative) OR (Postsurgical Pain) OR (Post-operative Pain) OR (Post operative Pain) OR (Post-operative Pains) OR (Postoperative Pain) OR (Postoperative Pain Chronic) OR (Pain Chronic Postoperative) OR (Chronic Postoperative Pain) OR (Chronic Post-surgical Pain) OR (Chronic Post surgical Pain) OR (Pain Chronic Post-surgical) OR (Post-surgical Pain Chronic) OR (Chronic Postsurgical Pain) OR (Chronic Postsurgical Pains) OR (Pain Chronic Postsurgical) OR (Postsurgical Pain Chronic) OR (Persistent Postsurgical Pain) OR (Pain Persistent Postsurgical) OR (Postsurgical Pain Persistent) OR (Post-operative Pain Chronic) OR (Pain Chronic Post-operative) OR (Post operative Pain Chronic) OR (Chronic Post-operative Pain) OR (Chronic Post operative Pain) OR (Postoperative Pain Acute) OR (Pain Acute Postoperative) OR (Acute Postoperative Pain) OR (Acute Post-operative Pain) OR (Acute Post operative Pain) OR (Post-operative Pain Acute) OR (Pain Acute Post-operative) OR (Post operative Pain Acute)) ([381.553](http://apps-webofknowledge.ez257.periodicos.capes.gov.br/summary.do?product=WOS&doc=1&qid=9&SID=7EGQ9RjqAZRCyhQcV4G&search_mode=AdvancedSearch&update_back2search_link_param=yes))

#4= TS=((Adrenal Cortex Hormones) OR Corticosteroids OR Corticosteroid OR Corticoids OR Corticoid OR (Glucocorticoids) OR Glucocorticoid OR (Glucocorticoid Effect) OR (Glucorticoid Effects) OR (Anti-Inflammatory Agents) OR (Anti Inflammatory Agents) OR (Antiinflammatory Agent) OR (Antiinflammatory Agents) OR Anti-Inflammatories OR (Anti Inflammatories) OR (Antiinflammatories) OR (Anti-Inflammatory Agent) OR (Anti Inflammatory Agent) OR (Agents Anti-Inflammatory) OR (Agents Anti Inflammatory) OR Dexamethasone OR Methylfluorprednisolone OR Betamethasone OR Flubenisolone OR Betadexamethasone OR (Methylprednisolone Acetate) OR Methylprednisolone OR Methylprednisolone-21-acetate OR (Methylprednisolone 21 acetate) OR Acetyl-Methylprednisolone OR (Acetyl Methylprednisolone) OR 6-Methylprednisolone OR (6 Methylprednisolone) OR Prednisone OR Dehydrocortisone OR delta-Cortisone OR Prednisolone OR Hidrocortisone OR cortisol OR (Anti-Inflammatory Agents Non-Steroidal) OR NSAID OR (Nonsteroidal Anti-Inflammatory Agent) OR (Agent Nonsteroidal Anti-Inflammatory) OR (Anti-Inflammatory Agent Nonsteroidal) OR (Nonsteroidal Anti Inflammatory Agent) OR NSAIDS OR (Antiinflammatory Agents Non Steroidal) OR (Antiinflammatory Agents Nonsteroidal) OR (Nonsteroidal Antiinflammatory Agents) OR (Non-Steroidal Anti-Inflammatory Agents) OR (Non Steroidal Anti Inflammatory Agents) OR (Nonsteroidal Anti-Inflammatory Agents) OR (Nonsteroidal Anti Inflammatory Agents) OR (Non-Steroidal Anti-Inflammatory Agent) OR (Agent Non-Steroidal Anti-Inflammatory) OR (Anti-Inflammatory Agent Non-Steroidal) OR (Non Steroidal Anti Inflammatory Agent) OR (Anti Inflammatory Agents Nonsteroidal) OR (Analgesics Anti-Inflammatory) OR (Anti-Inflammatory Analgesics) OR (Aspirin-Like Agents) OR (Aspirin Like Agents) OR (Aspirin-Like Agent) OR (Agent Aspirin-Like) OR (Aspirin Like Agent) OR (Cyclooxygenase 2 Inhibitors) OR (Cyclooxygenase 2 Inhibitor) OR (COX-2 Inhibitor) OR (COX 2 Inhibitor) OR Coxib OR Coxibs OR (COX-2 Inhibitors) OR (COX 2 Inhibitors) OR (COX2 Inhibitors) OR (COX2 Inhibitor) OR (Cyclooxygenase-2 Inhibitors) OR (Cyclooxygenase-2 Inhibitor) OR Celecoxib OR Ketoprofen OR diclofenac OR flurbiprofen OR Ibuprofen OR Indomethacin OR ketorolac OR lornoxicam OR (mefenamic acid) OR meloxicam OR naproxen OR piroxicam OR sulindac OR celecoxib OR valdecoxib OR lumiracoxib OR rofecoxib OR etoricoxib OR parecoxib OR etodolac OR Analgesics OR Anodynes OR (Analgesic Drugs) OR Analgesic OR (Analgesic Agents) OR (Antinociceptive Agents) OR (Agent Antinociceptive) OR Dipyrone OR Methamizole OR Metamizol OR Dipyronium OR Metamizole OR Acetaminophen OR Acetominophen OR Acetaminophen OR Acetamidophenol OR (Analgesics Opioid) OR (Opioid Analgesics) OR (Opioid Analgesic) OR Opioids OR Opioid OR (Opioid Partial Agonists) OR (Agonists Opioid Partial) OR (Full Opioid Agonists) OR tramadol OR codeine OR pentazocine OR tapentadol OR oxycodone) ([847.533](http://apps-webofknowledge.ez257.periodicos.capes.gov.br/summary.do?product=WOS&doc=1&qid=8&SID=7EGQ9RjqAZRCyhQcV4G&search_mode=AdvancedSearch&update_back2search_link_param=yes))

\# 3= TS=((Maxillofacial Surgery) OR (Oral Surgery) OR Exodontics OR (Third Molar) OR (Third Molars) OR (Wisdom Tooth) OR (Wisdom Teeth) OR (Impacted Tooth) OR (Impacted Teeth) OR (Dental Implant) OR (Dental Implants) OR (Unerupted Tooth) OR (Unerupted Teeth) OR (Supernumerary Tooth) OR (Supernumerary Teeth) OR (Fourth Molar) OR (Fourth Molars) OR (Bone Transplantation) OR (Bone Grafting) OR Autograft OR (Autologous Transplants) OR (Autologous Transplant) OR Autotransplants OR Autotransplant OR Allografts OR Allograft OR (Allogeneic Transplants) OR (Allogeneic Transplant) OR (Allogeneic Grafts) OR (Allogeneic Graft) OR Homografts OR Homograft OR (Homologous Transplants) OR (Homologous Transplant) OR Heterografts OR Heterograft OR Xenografts OR Xenograft) ([431.640](http://apps-webofknowledge.ez257.periodicos.capes.gov.br/summary.do?product=WOS&doc=1&qid=7&SID=7EGQ9RjqAZRCyhQcV4G&search_mode=AdvancedSearch&update_back2search_link_param=yes))

\# 2= TS=(Pulpitis OR Pulpitides OR (Endodontic Inflammation) OR (Endodontic Inflammations) OR endodontics OR endodontology OR (Dental Pulp Diseases) OR (Dental Pulp Disease) OR (root canal therapy) OR (root canal therapies) OR endodontic OR (periapical periodontitis) OR (Periapical Periodontitides) OR (Apical Periodontitides) OR (Apical Periodontitis) OR (Acute Nonsuppurative Periodontitides) OR (Acute Nonsuppurative Periodontitis) OR (dental pulp necrosis) OR (Dental Pulp Necroses) OR (Dental Pulp Necrosis) OR (Pulp Necroses) OR (Pulp Necrosis) OR (dental pulp gangrene) OR (dental pulp gangrenes) OR (Pulp Gangrene) OR (Pulp Gangrenes) OR (Pulp Mummification) OR (Pulp Mummifications) OR (Dental Pulp Autolysis) OR (Dental Pulp Autolyses) OR (nonvital tooth) OR (tooth devitalized) OR (tooth pulpless) OR (teeth pulpless) OR (teeth devitalized) OR (nonvital teeth) OR (teeth endodontically-treated) OR (teeth endodontically treated) OR (tooth endodontically-treated) OR (tooth endodontically treated) OR (granuloma periapical) OR (granulomas periapical) OR (periapical periodontitis chronic nonsuppurative) OR (periodontitis apical chronic nonsuppurative) OR (dental granulomas) OR (dental granuloma) OR (periodontitis apical suppurative) OR (abscess periapical) OR (abscesses periapical) OR (periapical periodontitides suppurative) OR (alveolar abscess apical) OR (abscesses apical alveolar) OR (dentoalveolar abscess apical) OR (abscesses apical dentoalveolar) OR Pulpectomy OR Pulpectomies OR (root canal preparation) OR (root canal preparations)) ([24.998](http://apps-webofknowledge.ez257.periodicos.capes.gov.br/summary.do?product=WOS&doc=1&qid=5&SID=7EGQ9RjqAZRCyhQcV4G&search_mode=AdvancedSearch&update_back2search_link_param=yes))

\# 1 TS=("systematic review" OR "systematic literature review" OR "systematic scoping review" OR "systematic narrative review" OR "systematic qualitative review" OR "systematic evidence review" OR "systematic quantitative review" OR "systematic meta-review" OR "systematic critical review" OR "systematic mixed studies review" OR "systematic mapping review" OR "systematic cochrane review" OR "systematic search") ([231.082](http://apps-webofknowledge.ez257.periodicos.capes.gov.br/summary.do?product=WOS&doc=1&qid=4&SID=7EGQ9RjqAZRCyhQcV4G&search_mode=AdvancedSearch&update_back2search_link_param=yes))

**Scopus (n=576 results)**

(Filtros de RS apenas em TITLE-ABS-KEY) excluindo indexados no medline e embase

( ( ( ALL ( pulpitis  OR  pulpitides  OR  ( endodontic  AND inflammation )  OR  ( endodontic  AND inflammations )  OR  endodontics  OR  endodontology  OR  ( dental  AND pulp  AND diseases )  OR  ( dental  AND pulp  AND disease )  OR  ( root  AND canal  AND therapy )  OR  ( root  AND canal  AND therapies )  OR  endodontic  OR  ( periapical  AND periodontitis )  OR  ( periapical  AND periodontitides )  OR  ( apical  AND periodontitides )  OR  ( apical  AND periodontitis )  OR  ( acute  AND nonsuppurative  AND periodontitides )  OR  ( acute  AND nonsuppurative  AND periodontitis )  OR  ( dental  AND pulp  AND necrosis )  OR  ( dental  AND pulp  AND necroses )  OR  ( dental  AND pulp  AND necrosis )  OR  ( pulp  AND necroses )  OR  ( pulp  AND necrosis )  OR  ( dental  AND pulp  AND gangrene )  OR  ( dental  AND pulp  AND gangrenes )  OR  ( pulp  AND gangrene )  OR  ( pulp  AND gangrenes )  OR  ( pulp  AND mummification )  OR  ( pulp  AND mummifications )  OR  ( dental  AND pulp  AND autolysis )  OR  ( dental  AND pulp  AND autolyses )  OR  ( nonvital  AND tooth )  OR  ( tooth  AND devitalized )  OR  ( tooth  AND pulpless )  OR  ( teeth  AND pulpless )  OR  ( teeth  AND devitalized )  OR  ( nonvital  AND teeth )  OR  ( teeth  AND endodontically-treated )  OR  ( teeth  AND endodontically  AND treated )  OR  ( tooth  AND endodontically-treated )  OR  ( tooth  AND endodontically  AND treated )  OR  ( granuloma  AND periapical )  OR  ( granulomas  AND periapical )  OR  ( periapical  AND periodontitis  AND chronic  AND nonsuppurative )  OR  ( periodontitis  AND apical  AND chronic  AND nonsuppurative )  OR  ( dental  AND granulomas )  OR  ( dental  AND granuloma )  OR  ( periodontitis  AND apical  AND suppurative )  OR  ( abscess  AND periapical )  OR  ( abscesses  AND periapical )  OR  ( periapical  AND periodontitides  AND suppurative )  OR  ( alveolar  AND abscess  AND apical )  OR  ( abscesses  AND apical  AND alveolar )  OR  ( dentoalveolar  AND abscess  AND apical )  OR  ( abscesses  AND apical  AND dentoalveolar )  OR  pulpectomy  OR  pulpectomies  OR  ( root  AND canal  AND preparation )  OR  ( root  AND canal  AND preparations ) ) )  OR  ( ALL ( ( maxillofacial  AND surgery )  OR  ( oral  AND surgery )  OR  exodontics  OR  ( third  AND molar )  OR  ( third  AND molars )  OR  ( wisdom  AND tooth )  OR  ( wisdom  AND teeth )  OR  ( impacted  AND tooth )  OR  ( impacted  AND teeth )  OR  ( dental  AND implant )  OR  ( dental  AND implants )  OR  ( unerupted  AND tooth )  OR  ( unerupted  AND teeth )  OR  ( supernumerary  AND tooth )  OR  ( supernumerary  AND teeth )  OR  ( fourth  AND molar )  OR  ( fourth  AND molars )  OR  ( bone  AND transplantation )  OR  ( bone  AND grafting )  OR  autograft  OR  ( autologous  AND transplants )  OR  ( autologous  AND transplant )  OR  autotransplants  OR  autotransplant  OR  allografts  OR  allograft  OR  ( allogeneic  AND transplants )  OR  ( allogeneic  AND transplant )  OR  ( allogeneic  AND grafts )  OR  ( allogeneic  AND graft )  OR  homografts  OR  homograft  OR  ( homologous  AND transplants )  OR  ( homologous  AND transplant )  OR  heterografts  OR  heterograft  OR  xenografts  OR  xenograft ) ) )  AND  ( ALL ( ( adrenal  AND cortex  AND hormones )  OR  corticosteroids  OR  corticosteroid  OR  corticoids  OR  corticoid  OR  ( glucocorticoids )  OR  glucocorticoid  OR  ( glucocorticoid  AND effect )  OR  ( glucorticoid  AND effects )  OR  ( anti-inflammatory  AND agents )  OR  ( anti  AND inflammatory  AND agents )  OR  ( antiinflammatory  AND agent )  OR  ( antiinflammatory  AND agents )  OR  anti-inflammatories  OR  ( anti  AND inflammatories )  OR  ( antiinflammatories )  OR  ( anti-inflammatory  AND agent )  OR  ( anti  AND inflammatory  AND agent )  OR  ( agents  AND anti-inflammatory )  OR  ( agents  AND anti  AND inflammatory )  OR  dexamethasone  OR  methylfluorprednisolone  OR  betamethasone  OR  flubenisolone  OR  betadexamethasone  OR  ( methylprednisolone  AND acetate )  OR  methylprednisolone  OR  methylprednisolone-21-acetate  OR  ( methylprednisolone  21  acetate )  OR  acetyl-methylprednisolone  OR  ( acetyl  AND methylprednisolone )  OR  6-methylprednisolone  OR  ( 6  methylprednisolone )  OR  prednisone  OR  dehydrocortisone  OR  delta-cortisone  OR  prednisolone  OR  hidrocortisone  OR  cortisol  OR  ( anti-inflammatory  AND agents  AND non-steroidal )  OR  nsaid  OR  ( nonsteroidal  AND anti-inflammatory  AND agent )  OR  ( agent  AND nonsteroidal  AND anti-inflammatory )  OR  ( anti-inflammatory  AND agent  AND nonsteroidal )  OR  ( nonsteroidal  AND anti  AND inflammatory  AND agent )  OR  NSAIDS  OR  ( antiinflammatory  AND agents  AND non  AND steroidal )  OR  ( antiinflammatory  AND agents  AND nonsteroidal )  OR  ( nonsteroidal  AND antiinflammatory  AND agents )  OR  ( non-steroidal  AND anti-inflammatory  AND agents )  OR  ( non  AND steroidal  AND anti  AND inflammatory  AND agents )  OR  ( nonsteroidal  AND anti-inflammatory  AND agents )  OR  ( nonsteroidal  AND anti  AND inflammatory  AND agents )  OR  ( non-steroidal  AND anti-inflammatory  AND agent )  OR  ( agent  AND non-steroidal  AND anti-inflammatory )  OR  ( anti-inflammatory  AND agent  AND non-steroidal )  OR  ( non  AND steroidal  AND anti  AND inflammatory  AND agent )  OR  ( anti  AND inflammatory  AND agents  AND nonsteroidal )  OR  ( analgesics  AND anti-inflammatory )  OR  ( anti-inflammatory  AND analgesics )  OR  ( aspirin-like  AND agents )  OR  ( aspirin  AND like  AND agents )  OR  ( aspirin-like  AND agent )  OR  ( agent  AND aspirin-like )  OR  ( aspirin  AND like  AND agent )  OR  ( cyclooxygenase  2  inhibitors )  OR  ( cyclooxygenase  2  inhibitor )  OR  ( cox-2  AND inhibitor )  OR  ( cox  2  inhibitor )  OR  coxib  OR  coxibs  OR  ( cox-2  AND inhibitors )  OR  ( cox  2  inhibitors )  OR  ( cox2  AND inhibitors )  OR  ( cox2  AND inhibitor )  OR  ( cyclooxygenase-2  AND inhibitors )  OR  ( cyclooxygenase-2  AND inhibitor )  OR  celecoxib  OR  ketoprofen  OR  diclofenac  OR  flurbiprofen  OR  ibuprofen  OR  indomethacin  OR  ketorolac  OR  lornoxicam  OR  ( mefenamic  AND acid )  OR  meloxicam  OR  naproxen  OR  piroxicam  OR  sulindac  OR  celecoxib  OR  valdecoxib  OR  lumiracoxib  OR  rofecoxib  OR  etoricoxib  OR  parecoxib  OR  etodolac  OR  analgesics  OR  anodynes  OR  ( analgesic  AND drugs )  OR  analgesic  OR  ( analgesic  AND agents )  OR  ( antinociceptive  AND agents )  OR  ( agent  AND antinociceptive )  OR  dipyrone  OR  methamizole  OR  metamizol  OR  dipyronium  OR  metamizole  OR  acetaminophen  OR  acetominophen  OR  acetaminophen  OR  acetamidophenol  OR  ( analgesics  AND opioid )  OR  ( opioid  AND analgesics )  OR  ( opioid  AND analgesic )  OR  opioids  OR  opioid  OR  ( opioid  AND partial  AND agonists )  OR  ( agonists  AND opioid  AND partial )  OR  ( full  AND opioid  AND agonists )  OR  tramadol  OR  codeine  OR  pentazocine  OR  tapentadol  OR  oxycodone ) )  AND  ( ALL ( preemptive  OR  preemptive  OR  ( pre  AND emptive )  OR  preventive  OR  premedication  OR  ( preoperative  AND care )  OR  ( care  AND preoperative )  OR  ( preoperative  AND procedure )  OR  ( procedures  AND preoperative )  OR  ( preoperative  AND period )  OR  ( period  AND preoperative )  OR  ( acute  AND pain )  OR  ( acute  AND pains )  OR  ( post-surgical  AND pain )  OR  ( pain  AND post-surgical )  OR  ( post  AND surgical  AND pain )  OR  ( pain  AND post-operative )  OR  ( pain  AND post  AND operative )  OR  ( postsurgical  AND pain )  OR  ( post-operative  AND pain )  OR  ( post  AND operative  AND pain )  OR  ( post-operative  AND pains )  OR  ( postoperative  AND pain )  OR  ( postoperative  AND pain  AND chronic )  OR  ( pain  AND chronic  AND postoperative )  OR  ( chronic  AND postoperative  AND pain )  OR  ( chronic  AND post-surgical  AND pain )  OR  ( chronic  AND post  AND surgical  AND pain )  OR  ( pain  AND chronic  AND post-surgical )  OR  ( post-surgical  AND pain  AND chronic )  OR  ( chronic  AND postsurgical  AND pain )  OR  ( chronic  AND postsurgical  AND pains )  OR  ( pain  AND chronic  AND postsurgical )  OR  ( postsurgical  AND pain  AND chronic )  OR  ( persistent  AND postsurgical  AND pain )  OR  ( pain  AND persistent  AND postsurgical )  OR  ( postsurgical  AND pain  AND persistent )  OR  ( post-operative  AND pain  AND chronic )  OR  ( pain  AND chronic  AND post-operative )  OR  ( post  AND operative  AND pain  AND chronic )  OR  ( chronic  AND post-operative  AND pain )  OR  ( chronic  AND post  AND operative  AND pain )  OR  ( postoperative  AND pain  AND acute )  OR  ( pain  AND acute  AND postoperative )  OR  ( acute  AND postoperative  AND pain )  OR  ( acute  AND post-operative  AND pain )  OR  ( acute  AND post  AND operative  AND pain )  OR  ( post-operative  AND pain  AND acute )  OR  ( pain  AND acute  AND post-operative )  OR  ( post  AND operative  AND pain  AND acute ) ) )  AND  ( TITLE-ABS-KEY ( ( systematic  AND review )  OR  ( systematic  AND literature  AND review )  OR  ( systematic  AND scoping  AND review )  OR  ( systematic  AND narrative  AND review )  OR  ( systematic  AND qualitative  AND review )  OR  ( systematic  AND evidence  AND review )  OR  ( systematic  AND quantitative  AND review )  OR  ( systematic  AND meta-review )  OR  ( systematic  AND critical  AND review )  OR  ( systematic  AND mixed  AND studies  AND review )  OR  ( systematic  AND mapping  AND review )  OR  ( systematic  AND cochrane  AND review )  OR  ( systematic  AND search  AND  review )  OR  ( systematic  AND integrative  AND review ) ) ) )  AND NOT  ( INDEX ( medline )  OR  INDEX ( embase ) )

**Virtual Health Library (n=503 results)**
(pulpitis OR pulpitides OR (endodontic inflammation) OR (endodontic inflammations) OR endodontics OR endodontology OR (dental pulp diseases) OR (dental pulp disease) OR (root canal therapy) OR (root canal therapies) OR endodontic OR (periapical periodontitis) OR (periapical periodontitides) OR (apical periodontitides) OR (apical periodontitis) OR (acute nonsuppurative periodontitides) OR (acute nonsuppurative periodontitis) OR (dental pulp necrosis) OR (dental pulp necroses) OR (dental pulp necrosis) OR (pulp necroses) OR (pulp necrosis) OR (dental pulp gangrene) OR (dental pulp gangrenes) OR (pulp gangrene) OR (pulp gangrenes) OR (pulp mummification) OR (pulp mummifications) OR (dental pulp autolysis) OR (dental pulp autolyses) OR (nonvital tooth) OR (tooth devitalized) OR (tooth pulpless) OR (teeth pulpless) OR (teeth devitalized) OR (nonvital teeth) OR (teeth endodontically-treated) OR (teeth endodontically treated) OR (tooth endodontically-treated) OR (tooth endodontically treated) OR (granuloma periapical) OR (granulomas periapical) OR (periapical periodontitis chronic nonsuppurative) OR (periodontitis apical chronic nonsuppurative) OR (dental granulomas) OR (dental granuloma) OR (periodontitis apical suppurative) OR (abscess periapical) OR (abscesses periapical) OR (periapical periodontitides suppurative) OR (alveolar abscess apical) OR (abscesses apical alveolar) OR (dentoalveolar abscess apical) OR (abscesses apical dentoalveolar) OR pulpectomy OR pulpectomies OR (root canal preparation) OR (root canal preparations) OR (maxillofacial surgery) OR (oral surgery) OR exodontics OR (third molar) OR (third molars) OR (wisdom tooth) OR (wisdom teeth) OR (impacted tooth) OR (impacted teeth) OR (dental implant) OR (dental implants) OR (unerupted tooth) OR (unerupted teeth) OR (supernumerary tooth) OR (supernumerary teeth) OR (fourth molar) OR (fourth molars) OR (bone transplantation) OR (bone grafting) OR autograft OR (autologous transplants) OR (autologous transplant) OR autotransplants OR autotransplant OR allografts OR allograft OR (allogeneic transplants) OR (allogeneic transplant) OR (allogeneic grafts) OR (allogeneic graft) OR homografts OR homograft OR (homologous transplants) OR (homologous transplant) OR heterografts OR heterograft OR xenografts OR xenograft) AND ((adrenal cortex hormones) OR corticosteroids OR corticosteroid OR corticoids OR corticoid OR (glucocorticoids) OR glucocorticoid OR (glucocorticoid effect) OR (glucorticoid effects) OR (anti-inflammatory agents) OR (anti inflammatory agents) OR (antiinflammatory agent) OR (antiinflammatory agents) OR anti-inflammatories OR (anti inflammatories) OR (antiinflammatories) OR (anti-inflammatory agent) OR (anti inflammatory agent) OR (agents anti-inflammatory) OR (agents anti inflammatory) OR dexamethasone OR methylfluorprednisolone OR betamethasone OR flubenisolone OR betadexamethasone OR (methylprednisolone acetate) OR methylprednisolone OR methylprednisolone-21-acetate OR (methylprednisolone 21 acetate) OR acetyl-methylprednisolone OR (acetyl methylprednisolone) OR 6-methylprednisolone OR (6 methylprednisolone) OR prednisone OR dehydrocortisone OR delta-cortisone OR prednisolone OR hidrocortisone OR cortisol OR (anti-inflammatory agents non-steroidal) OR nsaid OR (nonsteroidal anti-inflammatory agent) OR (agent nonsteroidal anti-inflammatory) OR (anti-inflammatory agent nonsteroidal) OR (nonsteroidal anti inflammatory agent) OR NSAIDS OR (antiinflammatory agents non steroidal) OR (antiinflammatory agents nonsteroidal) OR (nonsteroidal antiinflammatory agents) OR (non-steroidal anti-inflammatory agents) OR (non steroidal anti inflammatory agents) OR (nonsteroidal anti-inflammatory agents) OR (nonsteroidal anti inflammatory agents) OR (non-steroidal anti-inflammatory agent) OR (agent non-steroidal anti-inflammatory) OR (anti-inflammatory agent non-steroidal) OR (non steroidal anti inflammatory agent) OR (anti inflammatory agents nonsteroidal) OR (analgesics anti-inflammatory) OR (anti-inflammatory analgesics) OR (aspirin-like agents) OR (aspirin like agents) OR (aspirin-like agent) OR (agent aspirin-like) OR (aspirin like agent) OR (cyclooxygenase 2 inhibitors) OR (cyclooxygenase 2 inhibitor) OR (cox-2 inhibitor) OR (cox 2 inhibitor) OR coxib OR coxibs OR (cox-2 inhibitors) OR (cox 2 inhibitors) OR (cox2 inhibitors) OR (cox2 inhibitor) OR (cyclooxygenase-2 inhibitors) OR (cyclooxygenase-2 inhibitor) OR celecoxib OR ketoprofen OR diclofenac OR flurbiprofen OR ibuprofen OR indomethacin OR ketorolac OR lornoxicam OR (mefenamic acid) OR meloxicam OR naproxen OR piroxicam OR sulindac OR celecoxib OR valdecoxib OR lumiracoxib OR rofecoxib OR etoricoxib OR parecoxib OR etodolac OR analgesics OR anodynes OR (analgesic drugs) OR analgesic OR (analgesic agents) OR (antinociceptive agents) OR (agent antinociceptive) OR dipyrone OR methamizole OR metamizol OR dipyronium OR metamizole OR acetaminophen OR acetominophen OR acetaminophen OR acetamidophenol OR (analgesics opioid) OR (opioid analgesics) OR (opioid analgesic) OR opioids OR opioid OR (opioid partial agonists) OR (agonists opioid partial) OR (full opioid agonists) OR tramadol OR codeine OR pentazocine OR tapentadol OR oxycodone) AND (preemptive OR preemptive OR (pre emptive) OR preventive OR premedication OR (preoperative care) OR (care preoperative) OR (preoperative procedure) OR (procedures preoperative) OR (preoperative period) OR (period preoperative) OR (acute pain) OR (acute pains) OR (post-surgical pain) OR (pain post-surgical) OR (post surgical pain) OR (pain post-operative) OR (pain post operative) OR (postsurgical pain) OR (post-operative pain) OR (post operative pain) OR (post-operative pains) OR (postoperative pain) OR (postoperative pain chronic) OR (pain chronic postoperative) OR (chronic postoperative pain) OR (chronic post-surgical pain) OR (chronic post surgical pain) OR (pain chronic post-surgical) OR (post-surgical pain chronic) OR (chronic postsurgical pain) OR (chronic postsurgical pains) OR (pain chronic postsurgical) OR (postsurgical pain chronic) OR (persistent postsurgical pain) OR (pain persistent postsurgical) OR (postsurgical pain persistent) OR (post-operative pain chronic) OR (pain chronic post-operative) OR (post operative pain chronic) OR (chronic post-operative pain) OR (chronic post operative pain) OR (postoperative pain acute) OR (pain acute postoperative) OR (acute postoperative pain) OR (acute post-operative pain) OR (acute post operative pain) OR (post-operative pain acute) OR (pain acute post-operative) OR (post operative pain acute)) AND ( type_of_study:("systematic_reviews"))

**Epistemonikos (n=64 results)**

(title:(Pulpitis OR Pulpitides OR (Endodontic Inflammation) OR endodontics OR (Dental Pulp Disease) OR (root canal therapy) OR (root canal therapies) OR endodontic OR (periapical periodontitis) OR (Periapical Periodontitides) OR (Apical Periodontitides) OR (Apical Periodontitis) OR (Acute Nonsuppurative Periodontitides) OR (Acute Nonsuppurative Periodontitis) OR (Dental pulp necrosis) OR (Dental Pulp Necroses) OR (Pulp Necroses) OR (Pulp Necrosis) OR (Dental pulp gangrenes) OR (Pulp Gangrene) OR (Pulp Gangrenes) OR (granuloma periapical) OR (granulomas periapical) OR (periapical periodontitis chronic nonsuppurative) OR (periodontitis apical chronic nonsuppurative) OR (dental granulomas) OR (dental granuloma) OR (periodontitis apical suppurative) OR (abscess periapical) OR (abscesses periapical) OR (alveolar abscess apical) OR (abscesses apical alveolar) OR (dentoalveolar abscess apical) OR Pulpectomy OR Pulpectomies OR (root canal preparation) OR (Maxillofacial Surgery) OR (Oral Surgery) OR Exodontics OR (Third Molar) OR (Third Molars) OR (Wisdom Tooth) OR (Wisdom Teeth) OR (Impacted Tooth) OR (Impacted Teeth) OR (Dental Implant) OR (Dental Implants)) OR abstract:(Pulpitis OR Pulpitides OR (Endodontic Inflammation) OR endodontics OR (Dental Pulp Disease) OR (root canal therapy) OR (root canal therapies) OR endodontic OR (periapical periodontitis) OR (Periapical Periodontitides) OR (Apical Periodontitides) OR (Apical Periodontitis) OR (Acute Nonsuppurative Periodontitides) OR (Acute Nonsuppurative Periodontitis) OR (Dental pulp necrosis) OR (Dental Pulp Necroses) OR (Pulp Necroses) OR (Pulp Necrosis) OR (Dental pulp gangrenes) OR (Pulp Gangrene) OR (Pulp Gangrenes) OR (granuloma periapical) OR (granulomas periapical) OR (periapical periodontitis chronic nonsuppurative) OR (periodontitis apical chronic nonsuppurative) OR (dental granulomas) OR (dental granuloma) OR (periodontitis apical suppurative) OR (abscess periapical) OR (abscesses periapical) OR (alveolar abscess apical) OR (abscesses apical alveolar) OR (dentoalveolar abscess apical) OR Pulpectomy OR Pulpectomies OR (root canal preparation) OR (Maxillofacial Surgery) OR (Oral Surgery) OR Exodontics OR (Third Molar) OR (Third Molars) OR (Wisdom Tooth) OR (Wisdom Teeth) OR (Impacted Tooth) OR (Impacted Teeth) OR (Dental Implant) OR (Dental Implants))) AND (title:(Corticosteroids OR Corticosteroid OR Corticoids OR Corticoid OR Glucocorticoids OR Glucocorticoid OR (Anti-Inflammatory Agents) OR (Anti Inflammatory Agents) OR (Antiinflammatory Agent) OR (Antiinflammatory Agents) OR Anti-Inflammatories OR Antiinflammatories OR (Anti-Inflammatory Agents Non-Steroidal) OR NSAID OR (Nonsteroidal Anti-Inflammatory Agent) OR (Anti-Inflammatory Agent Nonsteroidal) OR (Nonsteroidal Anti Inflammatory Agent) OR NSAIDS OR (Antiinflammatory Agents Non Steroidal) OR (Antiinflammatory Agents Nonsteroidal) OR (Non-Steroidal Anti-Inflammatory Agents) OR (Nonsteroidal Anti Inflammatory Agents) OR (Non-Steroidal Anti-Inflammatory Agent) OR (Agent Non-Steroidal Anti-Inflammatory) OR (Anti-Inflammatory Agent Non-Steroidal) OR (Non Steroidal Anti Inflammatory Agent) OR (Anti Inflammatory Agents Nonsteroidal)) OR abstract:(Corticosteroids OR Corticosteroid OR Corticoids OR Corticoid OR Glucocorticoids OR Glucocorticoid OR (Anti-Inflammatory Agents) OR (Anti Inflammatory Agents) OR (Antiinflammatory Agent) OR (Antiinflammatory Agents) OR Anti-Inflammatories OR Antiinflammatories OR (Anti-Inflammatory Agents Non-Steroidal) OR NSAID OR (Nonsteroidal Anti-Inflammatory Agent) OR (Anti-Inflammatory Agent Nonsteroidal) OR (Nonsteroidal Anti Inflammatory Agent) OR NSAIDS OR (Antiinflammatory Agents Non Steroidal) OR (Antiinflammatory Agents Nonsteroidal) OR (Non-Steroidal Anti-Inflammatory Agents) OR (Nonsteroidal Anti Inflammatory Agents) OR (Non-Steroidal Anti-Inflammatory Agent) OR (Agent Non-Steroidal Anti-Inflammatory) OR (Anti-Inflammatory Agent Non-Steroidal) OR (Non Steroidal Anti Inflammatory Agent) OR (Anti Inflammatory Agents Nonsteroidal))) AND (title:(Preemptive OR Preemptive OR (Pre emptive) OR Preventive OR Premedication OR (Preoperative Care) OR (Period Preoperative) OR (Acute pain) OR (Post-surgical Pain) OR (Pain Post-operative) OR (Pain Post operative) OR (Postsurgical Pain) OR (Post-operative Pain) OR (Post operative Pain) OR (Postoperative Pain) OR (Acute Post-operative Pain) OR (Acute Post operative Pain)) OR abstract:(Preemptive OR Preemptive OR (Pre emptive) OR Preventive OR Premedication OR (Preoperative Care) OR (Period Preoperative) OR (Acute pain) OR (Post-surgical Pain) OR (Pain Post-operative) OR (Pain Post operative) OR (Postsurgical Pain) OR (Post-operative Pain) OR (Post operative Pain) OR (Postoperative Pain) OR (Acute Post-operative Pain) OR (Acute Post operative Pain)))
