# Supplementary material for: Effectiveness and Safety of Pretreatment Analgesics and Anti‐Inflammatory Drugs in Endodontic Pain Management: Overview of Systematic Reviews
Source: Int J Dent. 2026 Aug 9;2026:7258438. doi: 10.1155/ijod/7258438 (PMC13454743; doi:10.1155/ijod/7258438)
Supplement: Supplementary file 2 [file IJOD-2026-7258438-s002.docx]

**Supporting information 3 – Overlap of primary studies (RCT) in systematic reviews included and Corrected Covered Area (CCA)**

CCA corticosteroids and NSAID = 0,159 (very high overlap)

CCA corticosteroids = 0,300 (very high overlap)

CCA NSAID = 0,146 (high overlap)
