# Supplementary material for: Effectiveness and Safety of Pretreatment Analgesics and Anti‐Inflammatory Drugs in Endodontic Pain Management: Overview of Systematic Reviews
Source: Int J Dent. 2026 Aug 9;2026:7258438. doi: 10.1155/ijod/7258438 (PMC13454743; doi:10.1155/ijod/7258438)
Supplement: Supplementary file 3 [file IJOD-2026-7258438-s003.docx]

**Supporting Information 2: Excluded studies**

| **STUDIES** | **REASONS FOR EXCLUSION** |
| --- | --- |
| 1. ASHLEY, P. F. *et al*. Preoperative analgesics for additional pain relief in children and adolescents having dental treatment. Cochrane Database of Systematic Review, v. 2012, n. 9, 2012. | Other population |
| 2. ASHLEY, P. F. *et al*. Preoperative analgesics for additional pain relief in children and adolescents having dental treatment. Cochrane Database of Systematic Review, v. 2016, n. 8, 2016. | Other population |
| 3. BRIGNARDELLO-PETERSEN, R. Corticosteroids may reduce pain after endodontic treatment. Journal of the American Dental Association, v. 150, n. 3, E31-E31, 2019. | Other type of study |
| 4. CLARKE, R. *et al*. Single dose oral etoricoxib for acute postoperative pain in adult. Cochrane Database of Systematic Review, n. 2, 2009. | Other intervention |
| 5. CLARKE, R. SHEENA DERRY; R ANDREW MOORE. Single dose oral etoricoxib for acute postoperative pain in adults. Cochrane Database of Systematic Reviews, v. 2014, n. 5, 2014. | Other intervention |
| 6. COLLINS, S. L. et al. Oral ibuprofen and diclofenac in post-operative pain: a quantitative systematic review. European Journal of Pain, v. 2, n. 4, p. 285-291, 1998. | Other intervention |
| 7. HALL, P. E. *et al*. Single dose oral lornoxicam for acute postoperative pain in adults. Cochrane Database of Systematic Review, v. 2009, n. 4, out 2009. | Other intervention |
| 8. MACARIO, A; LIPMAN A.G. Ketorolac in the era of cyclo-oxygenase-2 selective nonsteroidal anti-inflammatory drugs: a systematic review of efficacy, side effects, and regulatory issues. Pain Medicine, v. 2, n. 4, p. 336-351, 2001. | Other population |
| 9. MOLL, R. *et al*. Single dose oral mefenamic acid for acute postoperative pain in adults. Cochrane Database of Systematic Reviews, v. 2017, v. 3, CD007553, 2011. | Other intervention |
| 10. MOORE, A. *et al*. Paracetamol with and without codeine in acute pain: A quantitative systematic review. Pain Apr, v. 70, n. 2-3, p. 193-201, 1997. | Other intervention |
| 11. SMITH L. A. *et al*. Single-dose ketorolac and pethidine in acute postoperative pain: systematic review with meta-analysis. British Journal of Anaesthesia. v. 84, n. 1, p. 48-58, 2000. | Other intervention |
| 12. TUPYOTA, P. *et al*. Supplementary techniques for pain control during root canal treatment of lower posterior teeth with irreversible pulpitis: A systematic review and meta-analysis. Aust Endod J, v. 44, n.1, p.14-25, April 2018. | Other outcome |
| 13. ZANJIR, M.; AZARPAZHOOH, A. Limited evidence suggests premedication with corticosteroids may help relieve postoperative endodontic pain in patients with symptomatic pulpitis undergoing single-visit root canal treatment. Journal of Evidence-Based Dental Practice, v. 20, n.1, p. 3, mar 2020. | Other type of study |
| 14. ZEMMEL, M.H. The role of COX-2 inhibitors in the perioperative setting: Efficacy and safety - A systematic review. AANA Journal, v.74, n.1, p.49-60, 2006. | Other type of study |
| 15. ZHOU, X. Y.; NANAYAKKARA, S. Corticosteroids can be effective introducing postoperative endodontic pain. Journal of Evidence-Based Dental Practice, v. 20, n.3, jun 2020. | Other type of study |
